# SCD2 Alleviates Diabetes‐Associated Cognitive Dysfunction by Improving Microglial Lipid Metabolism

**DOI:** 10.1111/cpr.70221

**Published:** 2026-04-30

**Authors:** Yang Yang, Juan He, Jing Zheng, Bing Shao, Lixin Shi, Miao Zhang

**Affiliations:** ^1^ Department of Endocrinology and Metabolism The Affiliated Hospital of Guizhou Medical University Guiyang Guizhou China; ^2^ Department of Neurology The Affiliated Hospital of Guizhou Medical University Guiyang Guizhou China; ^3^ Department of Endocrinology and Metabolism GuiQian International General Hospital Guiyang Guizhou China

**Keywords:** diabetes‐associated cognitive dysfunction, lipid droplet accumulation, microglia, neuroinflammation, oxidative phosphorylation, SCD2

## Abstract

The mechanisms underlying diabetes‐associated cognitive dysfunction (DACD) are not fully understood, and microglial metabolic dysfunction is emerging as a key contributor. This study investigates whether stearoyl‐CoA desaturase 2 (SCD2) alleviates cognitive impairment by modulating microglial lipid metabolism and function. Bioinformatics analysis of a single‐cell RNA‐seq dataset (GSE201644) identified SCD2 downregulation in diabetic (db/db) microglia. A T2D mouse model underwent hippocampal overexpression of SCD2 via AAV injection. In vitro, high glucose (HG)‐treated BV2 microglia‐like cells were subjected to SCD2 overexpression or oleic acid (OA) supplementation. Mitochondrial function (OCR, ATP, ETC complexes), lipid droplet accumulation (BODIPY, PLIN2), and inflammation (TNF‐α, IL‐6) were assessed. Cognitive behaviour (MWM, NOR) and neurophysiology (synaptic markers, neuronal survival) were evaluated. Diabetic microglia exhibited reduced SCD2 expression, impaired oxidative phosphorylation and lipid droplet accumulation (LDAM). SCD2 overexpression or OA rescued mitochondrial function, mitigated lipid droplet accumulation and attenuated inflammation. In vivo, hippocampal SCD2 overexpression attenuated neuroinflammation, preserved synaptic integrity and improved cognition in diabetic mice. SCD2 is essential for maintaining microglial lipid and mitochondrial homeostasis in diabetes. Restoring SCD2 function alleviates neuroinflammation and synaptic deficits, thereby rescuing cognitive impairment, highlighting its therapeutic potential for DACD.

## Graphical abstract

This study reveals a novel mechanism of microglial metabolic dysfunction in diabetic cognitive impairment. Defective SCD2 disrupts monounsaturated fatty acid (MUFA) metabolism, triggering mitochondrial oxidative phosphorylation dysfunction and leading to abnormal lipid droplet accumulation (marked by PLIN2). SCD2 overexpression (AAV‐SCD2) restores oxidative phosphorylation levels, clears lipid droplets, protects synapses and neuronal function and ultimately improves cognition. This study establishes the SCD2‐MUFA‐mitochondrial axis as a potential new therapeutic target for diabetic cognitive impairment.

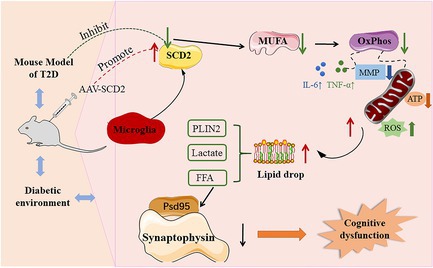

**Abbreviations**
AD: Alzheimer's disease
AOC: area over the curve
AP: anteroposterior
CEs: cholesterol esters
DACD: diabetes‐associated cognitive dysfunction
DM: diabetes mellitus
DV: dorsoventral
ELISA: enzyme‐linked immunosorbent assay
FC: free cholesterol
FFA: free fatty acids
GSK‐3β: glycogen synthase kinase‐3β
HG: high glucose
IF: immunofluorescence
LDAM: lipid droplet accumulation
MGL: monoacylglycerol
ML: mediolateral
MMP: mitochondrial membrane potential
mtDNA: mitochondrial DNA
MUFAs: monounsaturated fatty acids
OA: oleic acid
OxPhos: oxidative phosphorylation
PPARs: peroxisome proliferator‐activated receptors
qRT‐PCR: quantitative real‐time polymerase chain reaction
ROS: reactive oxygen species
SCD1: stearoyl‐CoA desaturase 1
SCD2: stearoyl‐CoA desaturase 2
SPF: specific pathogen‐free
T2DM: type 2 diabetes mellitus
TfamA: transcription factor A
TG: triacylglycerol
WB: western blotting

## 1 Introduction

Diabetes mellitus (DM) is a highly prevalent metabolic disease, and its association with cognitive dysfunction represents a major public health issue. Epidemiological studies have shown that nearly one‐third of diabetic patients experience varying degrees of cognitive impairment, characterized by reduced learning and memory abilities and a significantly increased risk of dementia, particularly among the elderly population with Type 2 diabetes (T2DM) [1, 2]. The underlying pathological mechanisms are complex, involving multiple interacting factors. Chronic hyperglycaemia and glucose fluctuations directly damage cognition‐associated brain regions such as the hippocampus by triggering oxidative stress, endothelial dysfunction and neuroinflammation [3]. Insulin resistance, on the other hand, accelerates Alzheimer's disease (AD)‐like pathology through activation of glycogen synthase kinase‐3β (GSK‐3β), promoting tau protein hyperphosphorylation and neurofibrillary tangle formation [4, 5]. Additionally, obesity is a significant risk factor for cognitive decline, primarily driven by adipose tissue dysfunction [6]. Despite these insights, specific therapeutic strategies targeting the molecular link between metabolic dysregulation and neurodegeneration remain limited.

Microglia act as immune sentinels in the central nervous system, and their metabolic status dynamically regulates neuroinflammation and neuronal survival. In diabetes and neurodegenerative diseases, pathological stimuli, such as Aβ plaques and tau protein aggregation, drive microglia to undergo metabolic reprogramming. This reprogramming is characterized by a shift from oxidative phosphorylation (OxPhos) toward glycolysis, accompanied by increased glucose uptake and the accumulation of lactate and lipids [7]. Consequently, microglia adopt a more pronounced pro‐inflammatory phenotype, marked by elevated secretion of TNF‐α, IL‐6 and exhibit impaired phagocytic function, thereby promoting neuronal degeneration [8]. A hallmark of this dysfunctional state is LDAM, which is closely linked to reduced mitochondrial OxPhos. Studies have shown that LDAM not only generates reactive oxygen species (ROS) and releases inflammatory factors, but also impairs Aβ clearance due to lysosomal dysfunction, establishing a vicious cycle that exacerbates pathology [9]. Therefore, targeting microglial metabolic homeostasis represents a promising strategy for intervening in diabetes‐related cognitive impairment.

Imbalance in fatty acid metabolism is a common feature of diabetes and neurodegenerative diseases. Within this context, the biological effects of monounsaturated fatty acids (MUFAs) are highly context‐dependent. Under physiological conditions, MUFAs can exert anti‐inflammatory effects by inhibiting NF‐κB and NLRP3 inflammasome activation through the activation of peroxisome proliferator‐activated receptors (PPARs) signalling [10]. However, in pathological states such as AD, MUFAs, notably OA produced by stearoyl‐CoA desaturase 1 (SCD1), can promote a pro‐inflammatory microglial phenotype and exacerbate neuroinflammation by disrupting the saturated/monounsaturated fatty acid ratio [11]. SCD2, a homologue of SCD1, is a key lipid‐metabolizing enzyme predominantly expressed in the brain and pancreas, where it catalyses the conversion of saturated fatty acids to MUFAs [12]. Its function displays spatiotemporal specificity: SCD2 supports the stratum corneum barrier and hepatic lipid synthesis during early development, whereas its activity is suppressed in adulthood [13]. Moreover, SCD2 activity can be modulated indirectly through pathways such as PPAR, which regulates lipid substrate availability and cellular energy homeostasis [14]. Recent evidence suggests that SCD2 may also influence the OxPhos efficiency by modulating mitochondrial phospholipid composition, particularly phosphatidylcholine [15]. However, its specific role in neuroimmune metabolism remains to be fully elucidated.

In diabetes, SCD2‐mediated MUFA metabolism may affect microglial function through two interconnected pathways. First, by regulating OxPhos, elevated SCD2 activity increases MUFA synthesis and modifies the mitochondrial membrane lipid composition, thereby maintaining electron transport chain efficiency and promoting ATP production [16]. This is consistent with the observation that the AMPK activator (e.g., AICAR) inhibits microglial inflammation by enhancing OxPhos [17]. Second, regarding lipid droplet homeostasis, MUFAs serve as core components of lipid droplets, and their accumulation is directly regulated by SCD2. Moderate lipid droplet formation buffers lipotoxicity, but excessive accumulation, such as in the APOE4 genotype, leads to impaired phagocytosis and increased ROS release in microglia. In diabetic models, hyperglycaemia and insulin resistance may contribute to the accumulation of microglial lipid droplets by up‐regulating SCD2 expression, whereas impaired OxPhos can further exacerbate inflammatory damage [18]. Notably, SCD2‐deficient mice exhibit impaired developmental lipid synthesis and defective barrier function [19], suggesting that tight regulation of SCD2 activity is crucial, and its dysregulation may contribute to lipid metabolic disturbances in diseases such as diabetes.

Although the clinical importance of diabetic cognitive impairment is widely recognized, there are fewer studies on whether reduced OxPhos in microglia is involved in diabetes‐related cognitive dysfunction through LDAM. This study aims to investigate whether SCD2 regulates microglial OxPhos through monounsaturated fatty acid metabolism, which in turn mediates microglial lipid droplet accumulation and contributes to cognitive dysfunction in diabetes. This study not only provides a new mechanistic explanation for diabetic cognitive impairment but also offers a theoretical basis for the development of therapeutic strategies targeting microglial metabolic homeostasis, which is of significant scientific and clinical value.

## 2 Materials and Methods

### 2.1 Bioinformatics Analysis

The diabetic cognitive impairment‐related dataset GSE201644 was downloaded from the GEO database (https://www.ncbi.nlm.nih.gov/geo/), containing samples from 1 db/db mouse (homozygous diabetic) and 1 db/m control mouse (heterozygous non‐diabetic). This dataset was originally generated by Ma et al. [20]. Single‐cell RNA sequencing data were analysed using the R package Seurat with the following quality control parameters: nFeature_RNA > 300; nCount_RNA > 500; nFeature_RNA < 8000; nCount_RNA < 80,000; and Percent MT < 20 for cell filtering. After data normalisation, dimensionality reduction was performed using the RunTSNE and RunUMAP functions for t‐SNE and UMAP visualisation, respectively. Cell clusters were annotated by identifying marker genes for various cell types, combined with automated cell type annotation using the ScType software.

Differential expression analysis between groups was performed using the FindMarkers function with the Wilcoxon rank‐sum test. Genes with an adjusted *p* value < 0.05 and |log2FC| > 0.25 were considered significant. The results were visualized through volcano plots generated with the ggplot2 R package. Gene expression patterns across groups were illustrated using the scCustomize package. Functional assessments were conducted by calculating Score, UCell and AUCell metrics via the AddModuleScore, AddModuleScore_UCell and AUCell_calcAUC functions, respectively. The results were subsequently visualized through heatmaps, dimensionality reduction plots, violin plots and boxplots.

### 2.2 Animals

6–8‐week‐old male C57BL/6 mice were obtained from Beijing SPF Biotechnology Co. Ltd. The animals were housed under specific pathogen‐free conditions with controlled temperature (21°C–26°C), humidity (30%–70%) and a 12‐h light/12‐h dark cycle. All mice had ad libitum access to food and water and underwent a 1‐week acclimatisation period before experimentation. All experimental protocols have been reviewed and approved by the Animal Ethics Committee of The Affiliated Hospital of Guizhou Medical University (No. 2403228).

### 2.3 Establishment of T2D Mouse Model

Mice were fed a high‐fat diet for 4 weeks, followed by intraperitoneal injection of streptozotocin (STZ, 60 mg/kg) on five consecutive days. Diabetes was confirmed by measuring tail vein blood glucose levels (≥ 16.7 mmol/L). Animals were maintained for an additional 7 weeks, with body weight and blood glucose monitored weekly.

C57BL/6 mice were randomly divided into four groups: (1) Control group: receiving an equivalent volume of saline via intraperitoneal injection. (2) T2D group: administered STZ (60 mg/kg). (3) T2D + AAV‐NC group: Received stereotaxic intracranial injection of SCD2 control vector. (4) T2D + AAV‐SCD2 group: Received stereotaxic intracranial injection of AAV‐SCD2. AAV‐SCD2 (10^12^ v.g./mL) or the control virus (AAV‐NC) was bilaterally injected into the hippocampus via stereotaxic injection. The injections were performed at three sites per hemisphere with the following coordinates relative to bregma: site 1 (anteroposterior, AP: −1.94 mm; mediolateral, ML: ±2.3 mm; dorsoventral, DV: −1.8 mm); site 2 (AP: −2.3 mm, ML: ±2.6 mm, DV: −2.0 mm); site 3 (AP: −2.54 mm, ML: ±2.9 mm, DV: −2.25 mm). A total of 2 μL virus suspension was delivered per site at a rate of 0.1 μL/min using a Hamilton syringe. The needle was retained for 5 min post‐injection before slow withdrawal to prevent reflux. All injections were performed at 4 weeks after T2D induction, and subsequent analyses were conducted 8 weeks post‐injection.

Mice were anaesthetised and transcardially perfused with pre‐cooled 0.01 M PBS to clear the blood, followed by perfusion with 4% paraformaldehyde for fixation. Whole brain tissues were rapidly removed. For BODIPY staining, the brain tissue was embedded in optimal cutting temperature (OCT) compound, snap‐frozen in liquid nitrogen and stored at −80°C until cryosectioning at 5 μm thickness. For H&E staining and immunofluorescence (IF) staining, the brain tissue was post‐fixed in 4% paraformaldehyde at 4°C for 24 h, dehydrated through graded ethanol, cleared in xylene and embedded in paraffin. Paraffin blocks were stored at room temperature and sectioned at 4 μm thickness.

### 2.4 Morris Water Maze Test

The Morris water maze test was performed using the KW‐MWM system (Nanjing Calvin Biotechnology) to evaluate spatial learning and memory in mice. During the acquisition phase (five consecutive days), mice were placed into the pool from four random starting points daily. The escape latency (EL, maximum 120 s) to locate the hidden platform was recorded, followed by a 10‐s platform stay. Each mouse underwent four training trials per day with 20‐min inter‐trial intervals. On Day 6, a probe trial was conducted with the platform removed. The time spent in the target quadrant and the number of platform crossings were recorded over 120 s. All behavioural data were collected and analysed using the KEMaze software system.

### 2.5 Novel Object Recognition Test

The novel object recognition test was performed using the KW‐SB system (Nanjing Calvin Biotechnology) to evaluate memory function in mice. First, mice underwent a 10‐min environmental adaptation period (without objects). Subsequently, two identical objects (A and B) were placed in the apparatus, and the mice's exploratory behaviour (contact or approaching within 2–3 cm) was recorded for 5 min. After 1 h, one of the objects was replaced with a novel object (AC or BC), and exploratory behaviour was recorded again for 5 min. All data were analysed using KEMaze software.

### 2.6 OGTT and IPITT Experiments

Both OGTT and IPITT experiments utilized a glucometer (Sinocare) for measuring blood glucose.

OGTT: Following an 8–10 h fast, mice received oral gavage of glucose (National Medicine; 1 g/kg body weight), and blood glucose was measured from the tail tip at 0, 30, 60, 90 and 120 min. The area over the curve (AOC) was calculated to assess glucose tolerance.

IPITT: After an 8–10 h fasting period, mice received an intraperitoneal injection of 0.75 U/kg insulin (Novo Nordisk), and blood glucose was measured at the same time points.

### 2.7 BODIPY Staining for Lipid Droplets

Frozen tissue sections were prepared as follows: fresh brain tissues were embedded in OCT compound and rapidly frozen in liquid nitrogen. Sections of 5 μm thickness were cut using a cryostat, mounted onto glass slides, and stored at −80°C until use. Before staining, sections were equilibrated at room temperature, fixed with 4% paraformaldehyde for 15 min, and washed three times with PBS. Sections were incubated with 2 μM BODIPY493/503 (Multifluorescence Biotech, P10051) at 37°C for 15 min. After washing three times with PBS, sections were stained with DAPI (Savior, G1012‐100ML) in the dark for 15 min. Following another three washes with PBS (5 min each), sections were mounted with anti‐fade mounting medium (Savior, G1401‐25 ML) and immediately examined under a fluorescence microscope.

### 2.8 Histological Staining

Mouse brain tissues were fixed in 4% paraformaldehyde, embedded in paraffin and sectioned at 4 μm. The tissue sections were deparaffinized and rehydrated. Sections were stained with haematoxylin (Savior, G1004) for 5 min and eosin (Savior, G1001) for 2 min. The slides were then dehydrated, cleared and cover‐slipped. Microscopic examination was performed to evaluate the structure and inflammatory response of the mouse brain tissues.

### 2.9 IF

IF detection of PLIN2, NeuN, CD16 and Iba1 expression in the mouse hippocampus and cortical tissues. Paraffin‐embedded sections (4 μm thickness) were deparaffinized and rehydrated. Slides were then permeabilized with 0.2% Triton X‐100 to enhance antibody penetration and blocked with 3% BSA for 30 min. For single IF staining, sections were incubated overnight at 4°C with the following primary antibodies: PLIN2 (1:100, Ab52356, Abcam), NeuN (1:100, GTX132974, GeneTex), CD16 (1:200, A23540, Abclonal), SCD2 (1:100, sc‐518034, Santa Cruz Biotechnology) and Iba1 (1:100, Ab178846, Abcam). For double IF co‐localisation analysis of PLIN2 and Iba1, sections were co‐incubated overnight at 4°C with PLIN2 (1:100, Ab52356, Abcam) and Iba1 (1:100, EPR16588, Abcam). After incubation, slides were incubated with FITC‐conjugated goat anti‐rabbit IgG secondary antibody (1:100, A0562, Beyotime) and Cy3‐conjugated goat anti‐rabbit IgG secondary antibody (1:100, A0516, Beyotime) at 37°C for 1 h. Following washing, sections were counterstained with DAPI and analysed under a fluorescence microscope. For quantitative analysis of hippocampal IF, images were captured from the CA1 and CA3 subregions based on the mouse brain stereotaxic coordinates. The dentate gyrus (DG) was excluded from all analyses. Mean fluorescence intensity was measured using ImageJ software, and the average value from all measured fields was calculated for each mouse for statistical analysis.

### 2.10 Isolation of Hippocampal Microglia by Flow Cytometry

Hippocampal tissues were rapidly dissected from euthanized mice and minced on ice. Tissue fragments were digested using a gentleMACS dissociator with Enzyme Mix 1 (Miltenyi Biotec) in the presence of transcription/translation inhibitors. After enzymatic dissociation, single‐cell suspensions were filtered through a 70‐μm strainer. Microglia were enriched via Percoll density gradient centrifugation (30%/37%/70%). Cells collected from the 37%–70% interface were subjected to Fc‐receptor blockade and stained with fluorescent antibodies against CD11b (1:100, 101211, Biolegend) and CD45 (1:100, 157213, Biolegend). CD11b^+^CD45^+^ microglia were isolated using a BD FACSAria III cell sorter, collected directly into TRIzol or RIPA lysis buffer for subsequent RNA and protein analyses.

### 2.11 Cell Culture and Treatment

Mouse microglia‐like cells (BV2) were purchased from Qingqi (Shanghai) Biotechnology Development Co. Ltd. Cells were cultured in DMEM medium supplemented with 10% fetal bovine serum (Gibco) and 1% penicillin–streptomycin. Standard culture conditions were maintained at 37**°C** in a humidified atmosphere with 5% CO_2_.

For experiments, BV2 cells were treated with plasma from T2D mice. To investigate the role of lipid components in T2D plasma, a subset of cells was co‐treated with T2D plasma and a lipid removal agent (LRA; Merck, 13358‐U). For transfection experiments, cells were transfected with Lipofectamine 8000 (4 μL) according to the manufacturer's instructions, with plasmids encoding SCD2 (2.5 μL) or a control vector, or with si‐TfamA (2.5 μL) or si‐NC (2.5 μL). After 24–48 h of transfection, cells were collected for subsequent experiments.

### 2.12 Detection of Intracellular ROS Levels

The ROS levels in cells from each group were measured using a ROS Assay Kit (S0033S, Beyotime). Cells in the logarithmic growth phase from each group were seeded in a 48‐well plate (three replicates per group) and cultured at 37°C for 24 h. After discarding the culture medium, 200 μL of 20 μM DCFH‐DA (diluted 1:500 in serum‐free medium) was added to each well, and the cells were incubated at 37°C in the dark for 30 min. The cells were then washed three times with PBS to remove any probe that did not enter the cells. Fluorescence images were captured immediately using an inverted fluorescence microscope (BZ‐X800, Keyence), and the fluorescence intensity was quantitatively analysed using ImageJ software.

### 2.13 Mitochondrial Membrane Potential (MMP) and ATP Measurement

MMP assay: The JC‐1 fluorescent probe method (C2006, Beyotime) was used. Cells were washed with PBS and incubated with 1 mL of JC‐1 working solution at 37°C in the dark for 20 min. Subsequently, cells were washed twice with pre‐chilled 1× JC‐1 buffer, and the red/green fluorescence ratio was detected using a flow cytometer (RMNNC‐3000, Agilent) to evaluate MMP changes.

ATP content detection: An ATP detection kit (BC0300, Solarbio) was used. Cells from each group were lysed in ice‐cold ATP extraction buffer (provided in the kit) supplemented with protease inhibitors. The lysates were centrifuged at 8000 × *g* for 10 min at 4°C, and the supernatant was collected. The absorbance at 340 nm was measured following the manufacturer's instructions, and the ATP concentration was calculated using a standard curve.

### 2.14 Evaluation of Mitochondrial ETC Activity

CheKine series kits (Complex I KTB1850, Complex II KTB1860, Complex III KTB1870, Complex IV KTB1880) provided by Abbkine were used. After homogenisation, mitochondria were sequentially centrifuged at 600 *g* (5 min) and 11,000 *g* (10 min) and resuspended to detect the activity of each complex: complex I (340 nm, Δ*A* = *A*_1_ − *A*_2_), complex II (605 nm, Δ*A* = *A*_1_ − *A*_2_), complex III (550 nm, Δ*A* = *A*_2_ − *A*_1_) and complex IV (550 nm, Δ*A* = *A*_1_ − *A*_2_). The enzyme activity was calculated by the value of absorbance change per unit time.

### 2.15 Cellular Energy Metabolism Assay

Cellular mitochondrial function (OCR) and glycolytic activity (ECAR) were assessed using the Agilent Seahorse XF analysis system. Cells were seeded at a density of 10^5^ cells/well in XF culture plates and cultured overnight at 37°C. The XF assay solution (containing 2 mM glutamine and 10 mM glucose) was changed to pH 7.4 and equilibrated without CO_2_ for 1 h at 37°C before the assay. Probe plates were hydrated overnight with calibration solution and then added to mitochondrial stress test drug (OCR assay) or glycolytic stress test drug (ECAR assay), respectively. Cellular oxygen consumption rate (OCR) and extracellular acidification rate (ECAR) were monitored in real time using a Seahorse XF Pro analyzer, and the data were analysed by Wave software to calculate parameters such as basal respiration, maximal respiration and glycolytic capacity.

### 2.16 Flow Cytometry Analysis

Lipid droplet assay: The content of cellular lipid droplets was analysed using the Lipid Droplet Green Fluorescence Detection Kit (C2053S, Beyotime). The cells in each group were digested by trypsin, washed with PBS and centrifuged at 400 × *g* for 5 min. The cell precipitates were resuspended with 0.5 mL of lipid droplet staining solution, incubated at room temperature and protected from light for 10–15 min, washed with PBS and then assayed on the machine. Fluorescence intensity was analysed using a Thermo Fisher Scientific Multiskan FC enzyme marker.

Activated microglia detection: IBA1 (1:100, 10904‐1‐AP, Proteintech) and CD68 (1:100, 14‐0688‐82, Thermo Fisher Scientific) expression was detected by IF double labelling. Cells were fixed with 4% paraformaldehyde, permeabilized with 0.5% Triton X‐100, and blocked with 5% goat serum. Primary antibodies were incubated overnight at 4°C, and FITC‐labelled goat anti‐rabbit IgG (A0562) and Cy3‐labelled goat anti‐mouse IgG (A0521) secondary antibodies were incubated away from light for 1 h. After washing with PBS, the proportion of activated microglia was observed and counted using a fluorescence microscope (Keyence, BZ‐X800).

### 2.17 Lactate Production Assay

A lactate test kit (A019‐2‐1, Nanjing Jiancheng) was used to determine cellular lactate production. The cells were collected in the logarithmic growth phase, centrifuged, and the supernatant was discarded. The cells were broken by ultrasonication with distilled water, and the supernatant was extracted by centrifugation at 8000 *g* for 10 min. The reaction system was prepared according to the instructions, and the absorbance values were measured at 530 nm using a Thermo Fisher Scientific Multiskan FC enzyme labeller. The lactic acid content of cells in each group was calculated by the standard curve, and three replicate wells were set up in each group.

### 2.18 Enzyme‐Linked Immunosorbent Assay (ELISA)

The levels of lipid metabolites, free fatty acids (FFA), triacylglycerol (TG), monoacylglycerol (MGL), free cholesterol (FC) and cholesterol esters (CEs) in microglia‐like cells, as well as the cytokines TNF‐α, IL‐4 and IL‐6, were quantitatively analysed by ELISA kit. For lipid metabolite measurement: Harvested cells were washed with PBS and resuspended in PBS to a concentration of approximately 1 × 10^6^ cells/mL. Cells were lysed by repeated freeze–thaw cycles, followed by centrifugation at 2000–3000 × *g* for 20 min at 4°C. The supernatant was carefully collected for analysis. For inflammatory factor measurements, cells were collected, washed with cold PBS, and lysed in PBS (150–200 μL per 10^6^ cells) by repeated freeze–thaw cycles or sonication. Lysates were centrifuged at 1500 × *g* for 10 min at 4°C, and the supernatant was used for the assay. Total protein concentration in cell lysates was determined using a BCA assay, and lipid metabolite levels were normalized to total protein content. Cytokine levels were expressed as pg/mL based on standard curves. The absorbance was measured at 405 nm by Multiskan FC (Thermo Fisher Scientific).

### 2.19 Western Blotting (WB)

Microglia‐like cells were lysed using RIPA lysis buffer (BL504A, Bio Sharp), followed by protein quantification using the Pierce BCA protein quantification kit (BL521A, Bio Sharp). Proteins were separated using 10% SDS‐PAGE and transferred to PVDF membranes using wet transfer. Membranes were closed with 5% skimmed milk powder for 2 h at room temperature. The primary antibodies were diluted to the target concentration in blocking solution, and the membranes were incubated with the antibody solution overnight at 4°C. Antibodies used included SCD2 primary antibody (1:1000, sc‐518034, Santa Cruz Biotechnology), Arg‐1 primary antibody (1:5000, ab91279, Abcam), YM‐1 primary antibody (1:5000, ab192029, Abcam), CD206 primary antibody (1:1000, 18704‐1‐AP, Proteintech), TFAM primary antibody (1:1000, A3173, Abclonal), PLIN2 primary antibody (1:1000, A6276, Abclonal), TOM20 primary antibody (1:1000, 11802‐1‐AP. Proteintech), VLCAD primary antibody (1:1000, ab155138, Abcam), iNOS primary antibody (1:1000, ab178945, Abcam), MHCII primary antibody (1:1000, ab180779, Abcam), CD16 primary antibody (1:1000, HY‐P81202, MCE), psd95 primary antibody (1:1000, 20665‐1‐AP, Proteintech), synaptophysin primary antibody (1:1000, ab192029, Abcam) and β‐actin primary antibody (1:20, 000, 66009‐1‐Ig, Proteintech). Next, the membranes were incubated with HRP‐conjugated secondary mouse IgG (1:5000, ab6789, Abcam) and secondary rabbit IgG (1:5000, ab6721, Abcam) at 37°C for 2 h. Finally, imaging was performed with the aid of an all‐in‐one chemiluminescent imaging system (ChampChemi 910, SINSAGE).

### 2.20 Quantitative Real‐Time Polymerase Chain Reaction (qRT‐PCR)

Total RNA was extracted from microglia‐like cells (G3013; Servicebio) using TRIzol reagent. RNA concentration and purity were assessed using a NanoDrop spectrophotometer (A260/A280 ratio > 1.8). cDNA was synthesized using the PrimeScript RT reagent Kit (RR037A, Takara) with 1 μg total RNA. cDNA was used as a template for PCR amplification according to the instructions for TaqMan One‐Step PrimeScript RT reagent Kit (RR037Q, Takara). Table 1 details information on all primers used in this experiment. In order to accurately assess the expression levels of SCD2 and TfamA mRNA in each group, all experimental data were analysed using GAPDH as an internal reference, three replicates of each reaction were performed, and quantitative analysis was performed using the 2^−∆∆CT^ method.

**TABLE 1:** Primers used in qRT‐PCR.

Gene	Forward sequence (5′‐3′)	Reverse sequence (5′‐3′)
β‐Actin (mouse)	GGCTGTATTCCCCTCCATCG	CCAGTTGGTAACAATGCCATGT
SCD2 (mouse)	GTCTGACCTGAAAGCCGAGAAG	GCAAGAAGGTGCTAACGCACAG
TFAM (mouse)	GAGGCAAAGGATGATTCGGCTC	CGAATCCTATCATCTTTAGCAAGC
si‐TfamA‐1 (mouse)	AGUUGAUGUUUGUAUAUAAUA	UUAUAUACAAACAUCAACUUA
si‐TfamA‐2 (mouse)	GGUGUAGCACAGUACAGAAGA	UUCUGUACUGUGCUACACCAG
HK2 (mouse)	GCCAGCCTCTCCTGATTTTAGTGT	GGGAACACAAAAGACCTCTTCTGG
mt‐nd1 (mouse)	CTAGCAGAAACAAACCGGGC	CCGGCTGCGTATTCTACGTT
mt‐nd2 (mouse)	CACGATCAACTGAAGCAGCAA	ACGATGGCCAGGAGGATAATT
mt‐cyb (mouse)	TTATCGCGGCCCTAGCAA	TAATCCTGTTGGGTTGTTTGATCC
mt‐co1 (mouse)	GAAGAGACAGTGTTTCATGTGGTGT	TCCTGGGCCTTTCAGGAATA
mt‐co2 (mouse)	CATCCCAGGCCGACTAAATC	TTTCAGAGCATTGGCCATAGAA
mt‐atp6 (mouse)	TGTGGAAGGAAGTGGGCAA	CCACTATGAGCTGGAGCCGT

### 2.21 Statistical Analysis

All results are expressed as mean ± standard deviation (SD). Each assay was performed three times. In this study, SPSS 16.0 statistical software was used to analyse the data. All continuous data were first assessed for normality using the Shapiro–Wilk test in conjunction with Q‐Q plots, and for homogeneity of variance using the Brown‐Forsythe test. Based on the test results: If both normality and homogeneity of variance were satisfied, unpaired two‐tailed *t*‐tests were used for comparisons between two groups, and ANOVA combined with Tukey's post hoc test was used for comparisons among multiple groups. If either condition was not satisfied, nonparametric tests were used (Mann–Whitney *U* test for two‐group comparisons; Kruskal–Wallis test combined with Dunn's post hoc test for multiple‐group comparisons). In addition, GraphPad Prism 8.0 was employed to complete the chart drawing. The criterion for statistical significance was set at *p* < 0.05.

## 3 Result

### 3.1 SCD2 and Oxidative Phosphorylation Were Downregulated in Microglia From Diabetic Mice

**FIGURE 1:**
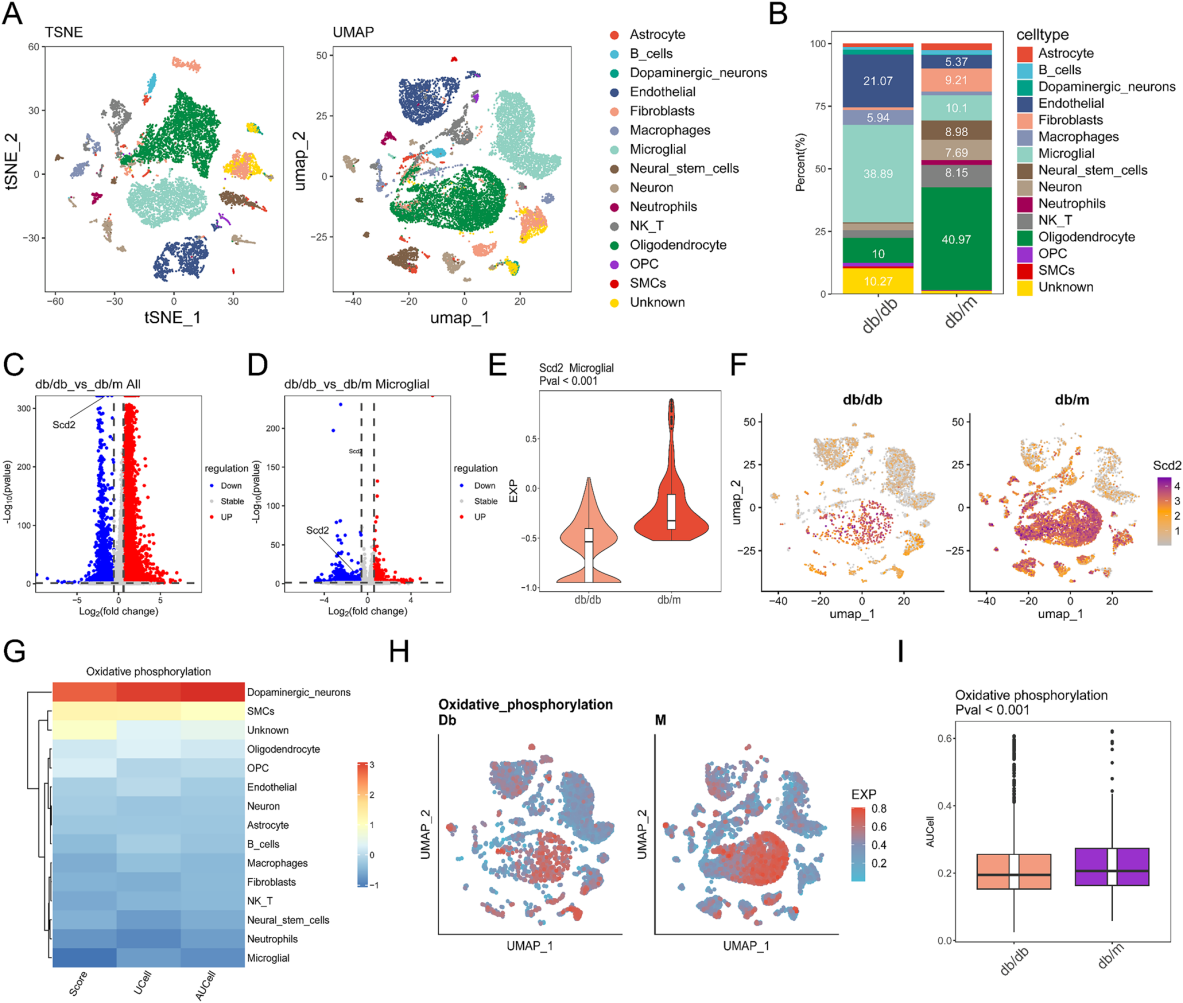
Downregulation of SCD2 and oxidative phosphorylation in microglia in diabetic cognitive dysfunction. Analysis was performed using dataset GSE201644 derived from mouse hippocampal tissue. (A) Distribution of different cell types in TSNE and UMAP dimensionality reduction plots. (B) Proportion of different cell types. (C) Volcano plot of inter‐group differential analysis for all cell types. (D) Volcano plot of inter‐group differential analysis for microglia. (E) Violin plot of inter‐group differential analysis of SCD2 in microglia. (F) Inter‐group differential analysis of the SCD2 gene across all cell types. (G) Heatmap of OxPhos activity in different cell types was evaluated using three scoring methods. (H) Distribution of OxPhos activity in different cells was assessed using AUCell score, stratified by genotype (db/m vs. db/db). (I) Comparison of OxPhos activity between db/m and db/db groups assessed by AUCell score.

Analysis of the public single‐cell dataset GSE201644 identified 26 distinct cell clusters in mouse hippocampal tissues (Figure 1A). Marker gene expression confirmed the identity of major cell types, with microglia constituting a significant proportion (Figure 1B). Cellular annotation reliability was validated by a heat map of marker gene expression (Figure S1A) and a heatmap of ScType cell type scores (Figure S1B). Whole‐cell differential analysis revealed extensive transcriptomic differences between the db/db diabetic group and db/m normal groups (Figure 1C).

To characterize SCD2 expression across different cell populations, we examined its distribution in various cell types. Violin plot analysis showed that SCD2 expression varied across cell types (Figure S2A). Further microglia‐specific analysis revealed significant downregulation of SCD2 expression in db/db microglia, as evidenced by both the volcano plot (Figure 1D) and the violin plot (Figure 1E). Notably, SCD2 was significantly downregulated across multiple cell types in diabetic mice (Figure 1F).

Subsequently, OxPhos activity was assessed using three independent scoring systems (AddModuleScore, UCell and AUCell): heatmap analysis showed that microglia in the db/db diabetic group had significantly lower OxPhos scores than the other cell types (Figure 1G); the AUCell score distribution, stratified by genotype, further confirmed that microglia exhibited the lowest OxPhos activity in the db/db group (Figure 1H). Moreover, comparison of activity distribution between groups showed that OxPhos activity of microglia in the db/db diabetic group was significantly lower than that in the db/m normal group (Figure 1I). To explore the relationship between SCD2 and OxPhos, correlation analysis revealed that SCD2 expression was positively correlated with OxPhos activity specifically in microglia (Figure S2B). Thus, microglia from db/db mice showed specific downregulation of SCD2 expression, accompanied by significantly reduced OxPhos activity.

### 3.2 SCD2 Expression Was Downregulated in Microglia of T2D Mice

**FIGURE 2:**
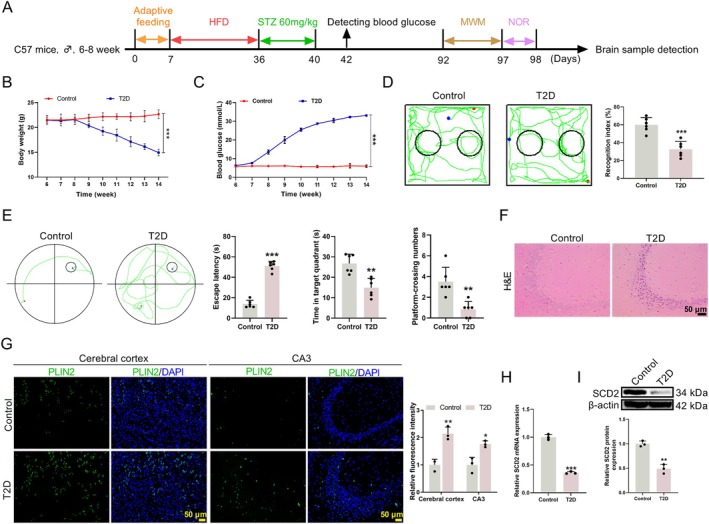
Reduced SCD2 expression in microglia of T2D mice. Construction of a T2D mouse model. (A) Timeline of experimental animals. (B) Changes in mouse body weight. (C) Blood glucose levels were measured from tail blood samples using a glucometer. (D) New object recognition test. (E) Morris water maze test. (F) H&E staining was utilized to observe morphological changes in hippocampal neurons. (G) IF detection of PLIN2 expression in the cerebral cortex and CA3 region. Flow cytometry sorting of microglia in the hippocampus of mice: (H, I) qRT‐PCR and WB were employed to assess SCD2 expression level in microglia. Data are presented as the mean ± SD; *n* = 6 (B–E), *n* = 3 (F–I). Statistical significance was determined by an unpaired two‐tailed Student's *t*‐test. *p < 0.05, **p < 0.01, ***p < 0.001 versus Control.

In the T2D mouse model (see Figure 2A for the experimental timeline), the T2D group exhibited a characteristic metabolic phenotype compared to the Control group: a persistent decrease in body weight (Figure 2B) and a significant increase in fasting blood glucose (Figure 2C). Further cognitive‐behavioural tests revealed cognitive dysfunction in the T2D group, as evidenced by a decreased novel object recognition index (Figure 2D) and a prolonged Morris water maze escape latency (Figure 2E). H&E staining revealed that hippocampal neurons were disorganized, with nuclear pyknosis and necrosis in the T2D group (Figure 2F). In addition, IF analysis further showed elevated PLIN2 expression, a marker of LDAM, in the cerebral cortex and CA3 region of T2D mice compared with the Control group (Figure 2G), suggesting abnormal lipid metabolism.

To further characterize the CA3 region, we analysed CA3‐related marker gene expression, which confirmed that cells in the CA3 region are predominantly microglia (Figure S2C). Given the importance of the CA3 region in cognitive function, we next investigated the spatial distribution of SCD2 in the hippocampus by IF staining. The results revealed that SCD2 expression was significantly decreased in the CA3 region of diabetic mice, with a similar but less pronounced reduction observed in the CA1 and DG regions (Figure S3).

To explore microglia‐specific changes, FACS sorting of hippocampal microglia was performed for molecular assays: qRT‐PCR and WB showed that SCD2 expression level was down‐regulated in the T2D group compared with the Control group (Figure 2H,I). Thus, SCD2 expression in hippocampal microglia was significantly down‐regulated in T2D model mice, accompanied by neuronal damage and cognitive dysfunction, with spatial changes predominantly in the CA3 region.

### 3.3 Overexpression of SCD2 Attenuated Oxidative Stress and Mitochondrial Dysfunction in High Glucose‐Exposed Microglia

To investigate whether lipid components in T2D plasma contribute to microglial activation, BV2 microglia‐like cells were treated with T2D mouse plasma in the presence or absence of LRA. Compared to the CON group, T2D plasma treatment significantly increased ROS levels (Figure S4A) and decreased ATP production (Figure S4B). This was accompanied by elevated secretion of pro‐inflammatory cytokines (TNF‐α and IL‐6) (Figure S4C), as well as downregulation of the protein expression levels of anti‐inflammatory markers (YM‐1, Arg‐1 and CD206) (Figure S4D). Notably, after removal of plasma lipids by LRA, all of the aforementioned molecular changes were significantly reversed (Figure S4A–D), indicating that lipid components in T2D plasma are critical for inducing microglial dysfunction and neuroinflammation.

**FIGURE 3:**
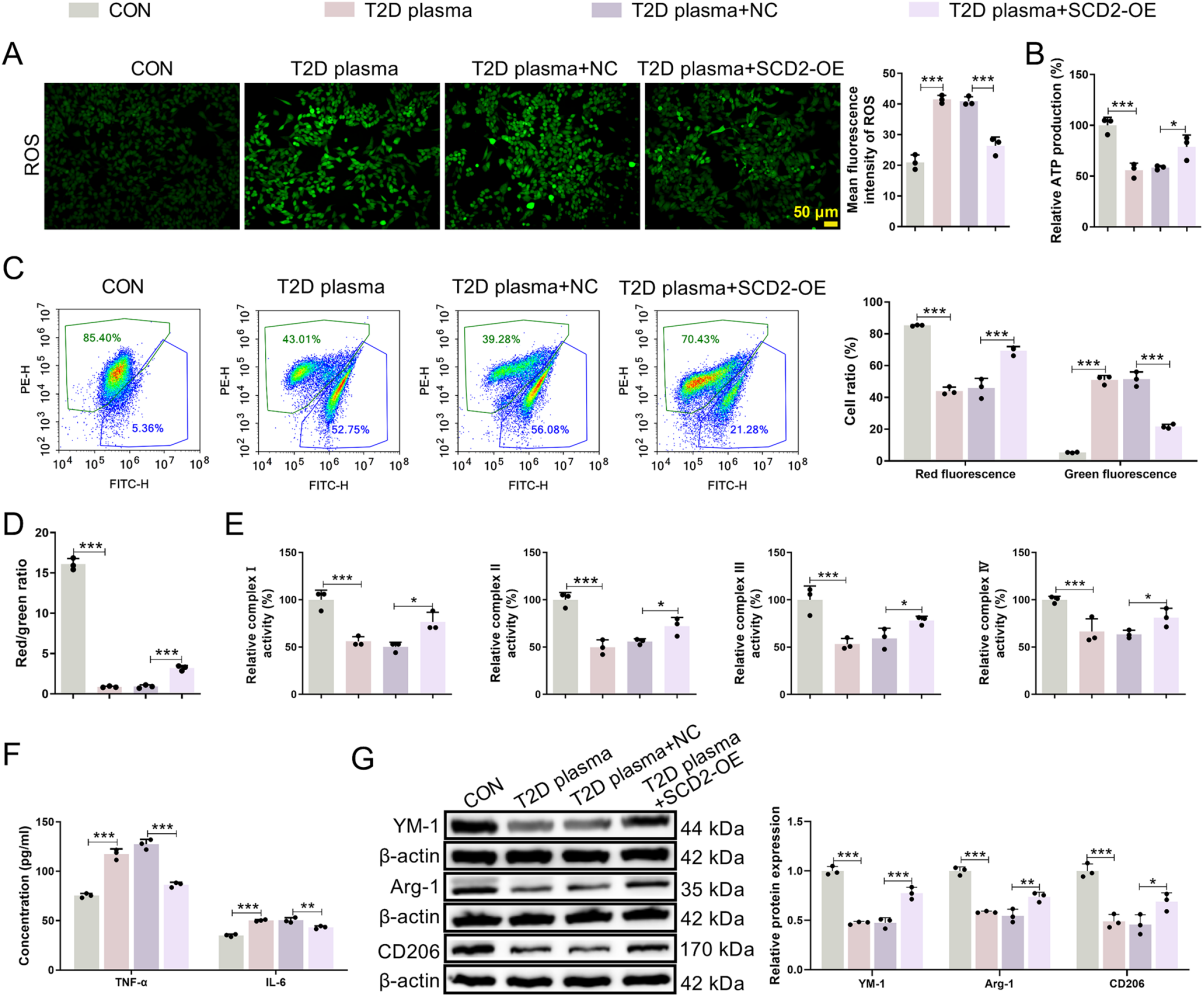
SCD2 overexpression mitigates high glucose‐induced oxidative stress and mitochondrial dysfunction in microglia‐like cells. BV2 cells (microglia‐like cells) were treated with plasma from T2D mice (hyperglycaemic plasma processed to remove EDTA and lipids). (A) ROS levels were detected using a ROS detection kit. (B) ATP levels were measured using an ATP content detection kit. (C, D) MMP was determined by flow cytometry. (E) Mitochondrial ETC activity was evaluated. (F) TNF‐α and IL‐6 levels were measured by ELISA. (G) The expression levels of anti‐inflammatory microglial phenotype markers YM‐1, Arg‐1, and CD206 were detected by WB. Data are presented as the mean ± SD; *n* = 3. Statistical analysis was performed by one‐way ANOVA followed by Tukey's post hoc test. *p < 0.05, **p < 0.01, ***p < 0.001 versus CON/T2D plasma + NC.

Based on the finding that lipid components drive microglial dysfunction, we further evaluated whether SCD2 overexpression could counteract these effects. BV2 microglia‐like cells were treated with T2D mouse plasma (hyperglycaemic condition) and subjected to SCD2 overexpression. The results of ROS and ATP level assays showed that the T2D plasma group had elevated ROS levels and reduced ATP levels compared to the CON group, whereas the SCD2‐OE treatment showed the opposite trend (Figure 3A,B). Analysis of MMP revealed that the T2D plasma group exhibited a significant decrease in red fluorescence intensity and a concomitant increase in green fluorescence intensity compared to the CON group (Figure 3C,D). Furthermore, ETC complex I, II, III and IV activities were decreased in the T2D plasma group, while the opposite trend was demonstrated after SCD2‐OE treatment (Figure 3E). ELISA revealed significantly higher levels of pro‐inflammatory factors TNF‐α and IL‐6 in T2D plasma compared with the CON, while the SCD2‐OE group showed decreased levels versus the NC group (Figure 3F). WB analysis further showed that YM‐1, Arg‐1 and CD206 protein expression levels were down‐regulated in the T2D plasma group, and the protein levels were increased after SCD2‐OE (Figure 3G). Therefore, SCD2 overexpression rescued mitochondrial dysfunction and attenuated inflammatory responses in HG‐treated microglia‐like cells.

### 3.4 Oxidative Phosphorylation Levels Regulated Lipid Droplet Accumulation in Microglia

**FIGURE 4:**
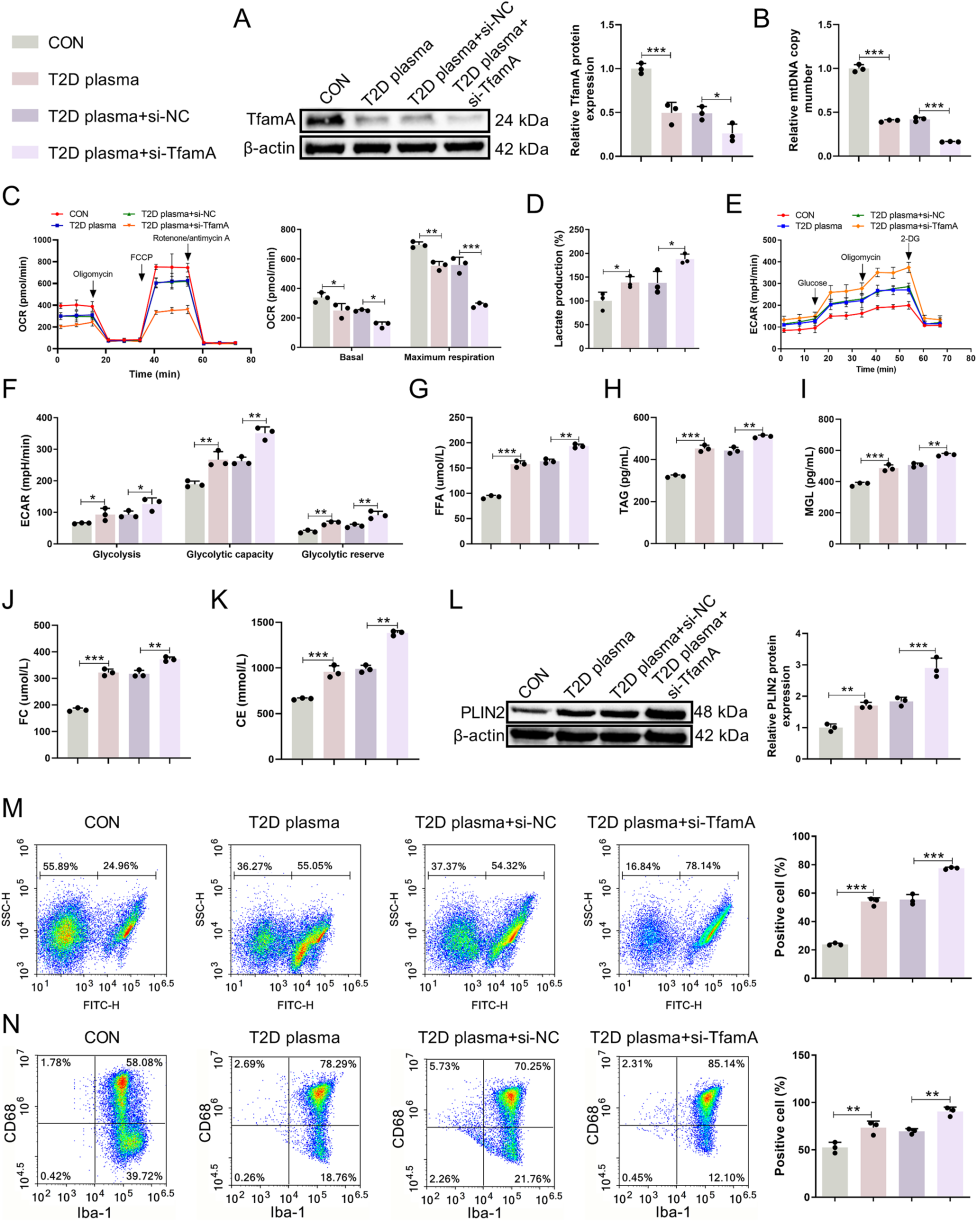
Oxidative phosphorylation levels in microglia mediate lipid droplet accumulation in microglia‐like cells. Construction of TfamA small interfering RNA (siRNA). (A) WB was applied to measure TfamA expression level. (B) qRT‐PCR detection of mtDNA copy number. (C) Real‐time oxygen consumption rate (OCR) was measured using a kit. (D) Lactate accumulation was detected using a lactate assay kit. (E, F) Extracellular acidification rate (ECAR) was measured using a kit. (G–K) ELISA detection of FFA, TAG, MGL, FC, and CE levels. (L) WB detection of PLIN2 expression level. (M) Flow cytometry was carried out to observe lipid droplets. (N) Flow cytometry was used to test activated microglia. Data are presented as the mean ± SD; *n* = 3. Statistical analysis was performed by one‐way ANOVA with Tukey's test (for normally distributed data) or the Kruskal–Wallis test with Dunn's test (for non‐normally distributed data). *p < 0.05, **p < 0.01, ***p < 0.001 versus CON/T2D plasma + si‐NC.

TfamA, a key regulator of mitochondrial DNA (mtDNA), encodes essential subunits of the OxPhos system. Depletion of TFAM results in mtDNA deficiency and impaired OxPhos function. In this study, we inhibited mitochondrial OxPhos by targeting TfamA with si‐TfamA. The efficiency of si‐TfamA was successfully validated (Figure S5A,B). qRT‐PCR and WB showed that, in comparison with the CON group, the T2D plasma group showed a significant decrease in TfamA expression, and si‐TfamA treatment showed a further decreasing trend (Figure 4A). qRT‐PCR for further detection of mtDNA copy number showed a decrease in mtDNA copy number, and the levels of mtDNA‐encoded transcripts for subunits of complexes I, III, IV and V were also significantly reduced in the si‐TfamA group (Figures 4B and S5C). Real‐time OCR was further reduced (Figure 4C), lactate accumulation was elevated (Figure 4D), and ECAR was elevated (Figure 4E,F) in the si‐TfamA group, suggesting enhanced glycolytic compensation. ELISA, WB and flow cytometry assays showed that following treatment with T2D plasma, FFA, TAG, MGL, FC and CE levels increased (Figure 4G–K); lipid droplet‐encapsulated protein PLIN2 expression level was elevated (Figure 4L), the number of lipid droplets was increased (Figure 4M), and the proportion of activated microglia was elevated (Figure 4N); while si‐TfamA treatment showed the same trend. Thus, si‐TfamA‐mediated inhibition of OxPhos exacerbated microglial lipid metabolism disorders, LDAM and inflammatory activation in a HG environment.

### 3.5 Monounsaturated Fatty Acid Metabolism Regulated Oxidative Phosphorylation Levels in Microglia‐Like Cells

**FIGURE 5:**
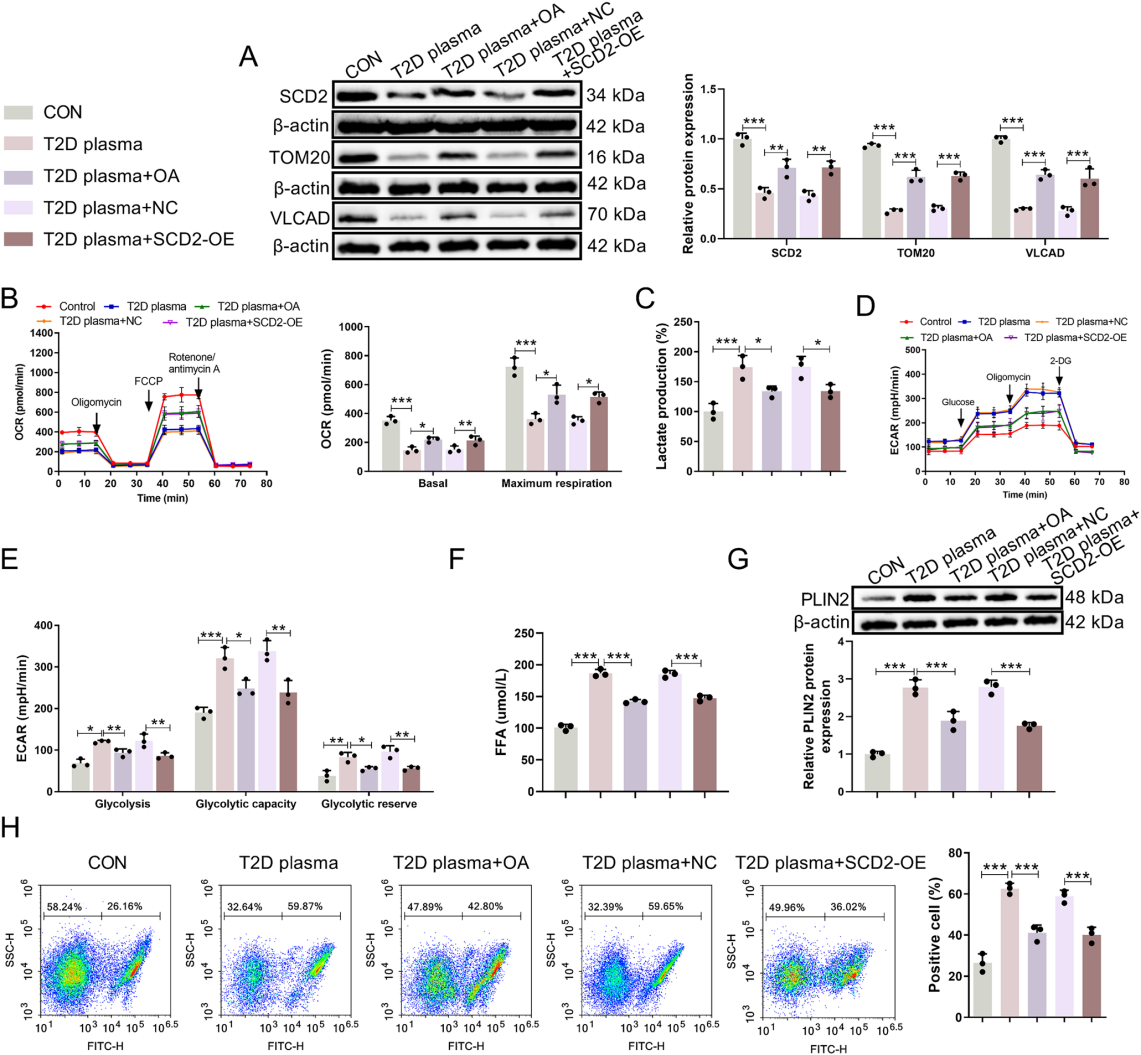
Monounsaturated fatty acid metabolism regulates oxidative phosphorylation levels in microglia‐like cells. BV2 cells were treated with OA. (A) The SCD2, TOM20, and VLCAD expression levels were examined by WB. (B) Real‐time OCR measured using a kit. (C) Lactate accumulation was detected using a lactate assay kit. (D, E) ECAR was evaluated using a kit. (F) ELISA was utilized to analyse FFA levels. (G) WB was conducted to assess PLIN2 expression level. (H) Flow cytometry analysis to observe lipid droplets. Data are presented as the mean ± SD; *n* = 3. Statistical analysis was performed by one‐way ANOVA with Tukey's test (for normally distributed data) or the Kruskal–Wallis test with Dunn's test (for non‐normally distributed data). *p < 0.05, **p < 0.01, ***p < 0.001 versus CON/T2D plasma/T2D plasma + NC.

OA was added to T2D plasma‐treated BV2 cells. WB showed that the expression of SCD2, TOM20 and VLCAD proteins was downregulated in the T2D plasma group and was elevated in OA or SCD2‐OE treatments (Figure 5A). Assessment of energy metabolism revealed decreased OCR and increased lactate levels and ECAR in the T2D plasma group, and the opposite trend was observed after the OA or SCD2‐OE treatment (Figure 5B–E). ELISA, WB and flow cytometry assays showed that FFA were increased in the T2D plasma group and decreased following treatment with OA or SCD2‐OE treatment (Figure 5F); the expression level of the lipid droplet‐encapsulating protein PLIN2 and the number of lipid droplets were elevated in the T2D plasma group, while OA or SCD2‐OE treatment downregulated its expression (Figure 5G,H). Thus, OA treatment reversed HG‐induced metabolic disorders and LDAM by activating SCD2‐mitochondrial function, and the effect was consistent with SCD2 overexpression.

### 3.6 SCD2 Overexpression Attenuated Microglial Pro‐Inflammatory Activation and Mitochondrial Dysfunction in T2D Mice

**FIGURE 6:**
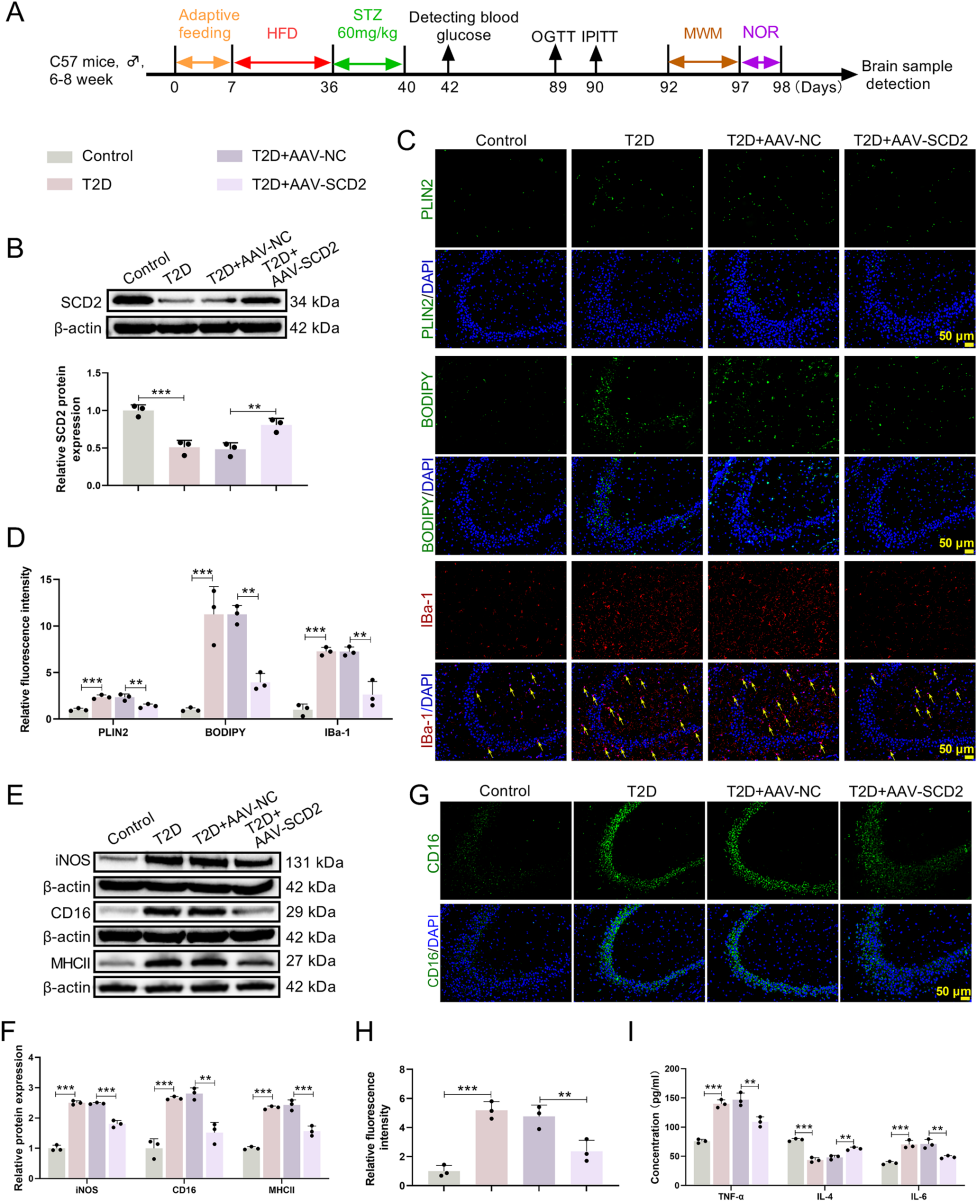
SCD2 overexpression inhibits the pro‐inflammatory phenotype in T2D mice and microglia. A T2D mouse model was constructed. At 4 weeks after T2D induction, AAV‐SCD2 was stereotaxically injected into the hippocampus. Hippocampal microglia were isolated from brain tissue 8 weeks post‐injection for analysis. (A) Timeline of experimental animals. (B) WB detection of SCD2 expression in microglia. (C, D) Coronal brain sections stained with IF for hippocampal PLIN2, BODIPY and IBa‐1 to detect lipid droplets. In panel (C), white arrows indicate representative Iba‐1‐positive microglia. (E, F) WB was performed to assess the expression levels of iNOS, MHC II, and CD16 in the hippocampus. (G, H) IF detection of CD16 expression. (I) ELISA measurement of TNF‐α, IL‐6 and IL‐4 levels. Data are presented as the mean ± SD; *n* = 3. Statistical analysis was performed by one‐way ANOVA followed by Tukey's post hoc test. **p < 0.01, ***p < 0.001 versus Control/T2D + AAV‐NC.

To investigate the effect of SCD2 on neuroinflammation in diabetic cognitive dysfunction, AAV‐SCD2 was injected into the hippocampus of T2D mice (see Figure 6A for the experimental timeline). WB analysis confirmed that SCD2 protein expression level was reduced in the T2D group, and it was elevated by AAV‐SCD2 treatment (Figure 6B). Immunostaining further showed that the fluorescence intensity of PLIN2, BODIPY and IBa‐1 was enhanced in the T2D group and was reduced by AAV‐SCD2 treatment (Figure 6C,D). To further validate that PLIN2 signalling is primarily localized within microglia, we employed IF co‐staining of PLIN2 and IBa‐1. The results demonstrated that PLIN2 and IBa‐1 exhibited distinct colocalisation, and AAV‐SCD2 treatment attenuated both IBa‐1 and PLIN2 fluorescence signals in T2D mice (Figure S6). WB results showed that the protein levels of iNOS, MHC II and CD16 were elevated in the T2D‐treated group; however, these protein expression levels were significantly reduced after AAV‐SCD2 treatment (Figure 6E,F). IF assay also confirmed the reduced immunoreactivity of CD16 after AAV‐SCD2 treatment (Figure 6G,H). ELISA analysis revealed that T2D treatment significantly elevated the levels of pro‐inflammatory cytokines (TNF‐α and IL‐6) and decreased the anti‐inflammatory cytokine IL‐4 in hippocampal tissues. These effects were reversed by AAV‐SCD2 treatment (Figure 6I).

To directly assess mitochondrial function in purified microglia, we isolated hippocampal microglia from each experimental group. Consistent with the findings in BV2 cells, T2D microglia exhibited impaired oxidative phosphorylation and lipid metabolism, as evidenced by decreased OCR and ATP levels, increased lactate accumulation and ECAR, elevated lipid metabolite levels (FFA, TAG, MGL, FC, CE), and upregulated PLIN2 protein expression level. Notably, all these abnormalities were reversed by AAV‐SCD2 treatment (Figure S7A–K). These results suggest that SCD2 overexpression attenuated the proinflammatory activation and mitochondrial dysfunction of microglia in the hippocampal region of T2D, thereby alleviating neuroinflammation.

### 3.7 SCD2 Overexpression Rescued Synaptic Protein Loss and Improved Neuronal Density in T2D Mice

**FIGURE 7:**
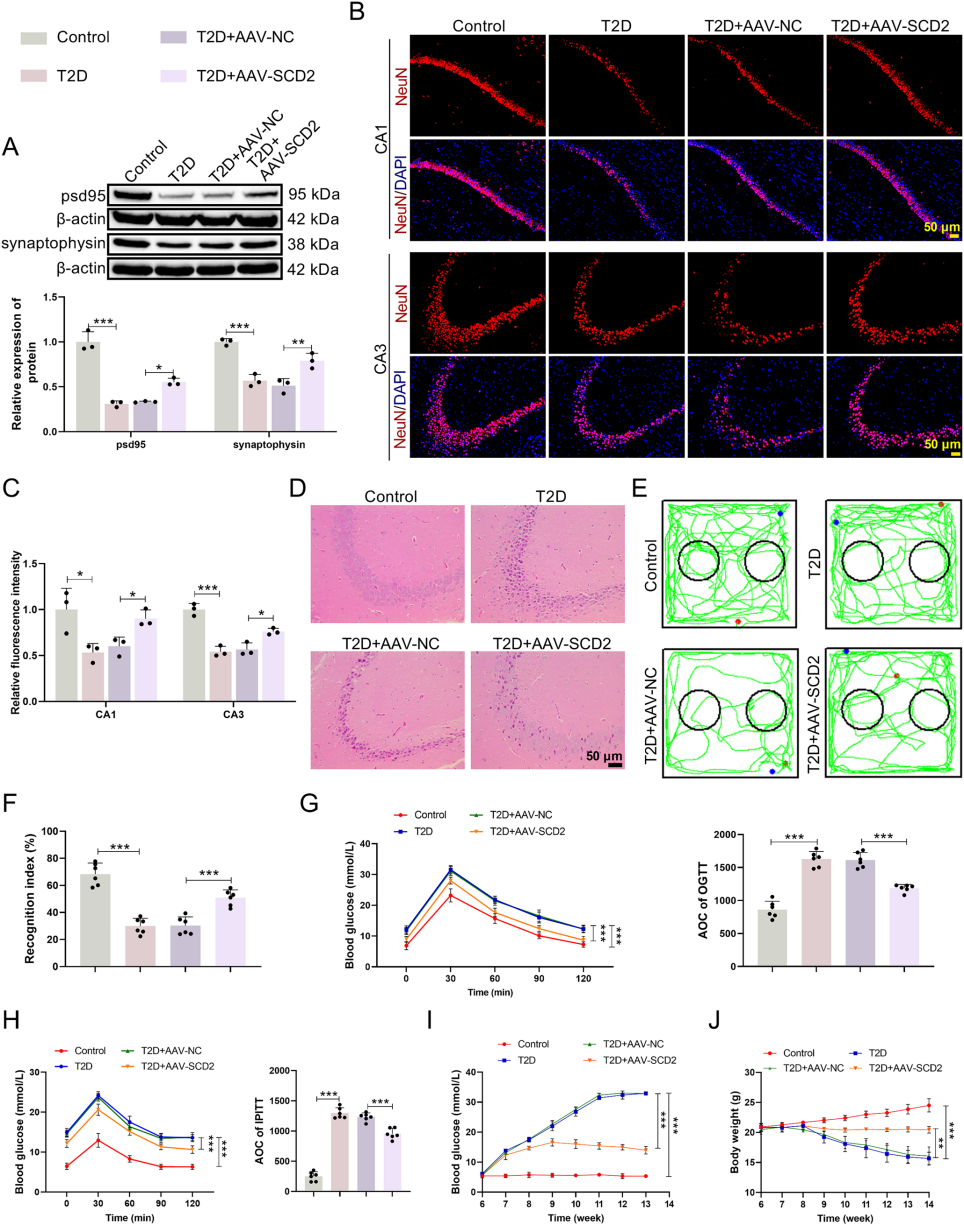
SCD2 overexpression rescues synaptic protein loss and improves neuronal density. (A) WB detection of synaptic markers PSD95 and synaptophysin expression levels. (B, C) Fluorescence staining was used to detect NeuN in the CA1 and CA3 regions of the hippocampus. (D) H&E staining was employed to observe morphological changes in hippocampal neurons. (E, F) New object recognition test. (G) Oral glucose tolerance test (OGTT) curve and AOC area measurement. (H) Intraperitoneal insulin tolerance test (IPITT) curve and AOC area measurement. (I) Blood glucose levels were measured from tail blood samples using a glucometer. (J) Changes in mouse body weight. Data are presented as the mean ± SD; *n* = 3 (A‐D), *n* = 6 (E–J). Statistical analysis was performed by one‐way ANOVA followed by Tukey's post hoc test. *p < 0.05, **p < 0.01, ***p < 0.001 versus Control/T2D + AAV‐NC.

To assess the neuroprotective effects of SCD2, we performed the injection of AAV‐SCD2 in the hippocampal region of T2D mice and isolated brain hippocampal microglia. WB analysis showed that psd95 and synaptophysin were significantly reduced in the T2D group, and AAV‐SCD2 restored the expression levels of these synaptic markers in hippocampal tissue (Figure 7A). NeuN IF staining revealed that T2D treatment significantly reduced neuronal density in hippocampal CA1 and CA3 subregions compared to the control group. However, AAV‐SCD2 treatment markedly improved neuronal density (Figure 7B,C). H&E staining revealed that hippocampal neurons in the control group exhibited regular arrangement, with round and translucent nuclei. In contrast, the T2D group displayed irregular neuronal morphology, nuclear pyknosis and necrosis. These diabetes‐induced histopathological changes were attenuated by AAV‐SCD2 treatment (Figure 7D). Novel object recognition test further demonstrated that the cognitive ability of AAV‐SCD2 mice was enhanced compared with the AAV‐NC group (Figures 7E,F and S8A,B). Metabolic profiling analysis confirmed that AAV‐SCD2 treatment decreased the AOC for OGTT and IPITT, and reduced blood glucose levels, while increasing body weight (Figure 7G–J), suggesting AAV‐SCD2 treatment improved glucose homeostasis. Thus, AAV‐SCD2 effectively rescued synaptic protein expression, preserved neuronal survival and improved cognitive function in T2D mice.

## 4 Discussion

In this study, we revealed the central regulatory role of SCD2 in DACD through multidimensional experiments. Specifically, we identified and validated for the first time that SCD2 significantly improves OxPhos levels in microglia by regulating monounsaturated fatty acid metabolism, thereby attenuating LDAM and neuroinflammation. Although SCD2 has been shown to function in other tissues, the present study revealed its importance in the central nervous system. This finding not only addresses a gap in the existing literature but also provides a new theoretical basis for understanding the pathological mechanisms of DACD [21].

Firstly, through bioinformatics analysis and in vitro and in vivo experiments, we clarified that SCD2 expression is significantly downregulated in microglia of diabetic mice, accompanied by reduced OxPhos activity, accumulation of lipid droplets, and cognitive decline. SCD2 deficiency has been shown to promote a pro‐inflammatory state in microglia, resulting in increased secretion of inflammatory factors, including IL‐1β and TNF‐α [22]. In vitro experiments have demonstrated that SCD2 overexpression in a high‐glycemia environment reverses mitochondrial dysfunction in microglia, inhibits inflammatory factor secretion, and reduces LDAM. Mechanistically, SCD2 overexpression likely increases the cellular pool of MUFAs, favouring the synthesis and storage of TG within lipid droplets over the accumulation of cytotoxic saturated lipid species [12]. SCD2 overexpression enhances mitochondrial membrane fluidity and stabilizes ETC function by increasing MUFA levels, thereby reducing ROS production. These results suggest that SCD2 may alleviate diabetes‐induced neuroinflammation and cognitive impairment by directly or indirectly modulating mitochondrial function and maintaining microglia homeostasis.

Beyond its metabolic role, SCD2 possesses broader physiological functions in the brain. The SCD family is involved in fundamental neurobiological processes, including neuronal development, synaptic plasticity and myelination. Inhibition of SCDs effectively improves synaptic and cognitive impairments in AD mouse models [23]. In multiple system atrophy, dysregulation of SCD is associated with reduced expression of synuclein proteins such as α‐synuclein, which may contribute to cognitive deficits [24]. Furthermore, SCD2‐derived MUFAs are key components of myelin lipids, and an increase in their abundance is closely linked to enhanced myelination [25]. These findings suggest that SCD2 may exert neuroprotective effects not only through metabolic regulation of microglia but also by directly supporting neuronal structure and function. Therefore, the downregulation of SCD2 observed in diabetic microglia may reflect a heightened susceptibility of the central nervous system to lipid imbalance, underscoring a mechanistic link to the pathogenesis of other cognitive disorders such as AD and vascular dementia.

Microglia play a critical role in cognitive dysfunction‐related diseases, including AD, Parkinson's disease (PD) and multiple sclerosis (MS) [26]. In AD, for instance, microglia recognize and phagocytose Aβ via pattern recognition receptors, exerting neuroprotective effects in the early stage of the disease. However, as the disease progresses, sustained Aβ stimulation leads to microglial dysfunction, characterized by decreased Aβ clearance capacity, excessive release of pro‐inflammatory factors, and abnormally enhanced synaptic phagocytosis, thereby accelerating tau protein propagation and cognitive decline [8]. Moreover, under pathological stimulation, microglia undergo metabolic reprogramming, shifting from oxidative phosphorylation to glycolysis, concomitant with lipid droplet accumulation and a pro‐inflammatory phenotype [27]. This LDAM state has been observed in both aging and AD models and is closely associated with impaired phagocytosis, increased ROS production, and neurotoxicity [28]. In the context of diabetes, chronic hyperglycaemia and insulin resistance further exacerbate microglial dysfunction, leading to persistent neuroinflammation and synaptic impairment [29]. These findings are highly consistent with our results, demonstrating that restoration of SCD2 expression in microglia reverses LDAM and inflammatory activation, thereby protecting neuronal integrity. These outcomes highlight the therapeutic potential of targeting microglial metabolism in cognitive disorders, including but not limited to diabetes.

Accumulating evidence indicates that lipid metabolism dysregulation is a critical pathological bridge linking diabetes and neurodegenerative diseases [30]. In the brain, lipid homeostasis is essential for membrane integrity, synaptic function and signal transduction [31]. More recently, lipid droplets have been recognized as key mediators of neuroinflammation and neurodegeneration [32]. For instance, in AD, the APOE4 genotype promotes microglial lipid accumulation and exacerbates Aβ pathology [33]. Notably, mitochondria‐associated membranes, the key contact sites between the endoplasmic reticulum and mitochondria, directly impair mitochondrial function when their lipid metabolism is disrupted, thereby aggravating neurodegeneration [34]. This aligns with our findings: hyperglycaemia and dyslipidaemia drive microglial lipid droplet formation through SCD2 downregulation, and these lipid‐laden microglia exhibit mitochondrial dysfunction and secrete pro‐inflammatory cytokines, creating a neurotoxic environment that accelerates cognitive decline. Our work highlights SCD2 as a central hub connecting lipid metabolism and microglial function and suggests that restoring SCD2 activity may have broad therapeutic implications for diseases characterized by lipid dysregulation and neuroinflammation.

Mechanistic studies have found that inhibition of TfamA exacerbates OxPhos defects and lipid metabolism disturbances, whereas OA intervention or SCD2 overexpression improves these phenotypes by activating the SCD2‐mitochondrial pathway. It has been shown that TfamA knockdown or deletion results in reduced mtDNA copy number, decreased expression of the ETC complex, and a significant reduction in ATP synthesis [35]. OA, the primary product of SCD2, may enhance SCD2 activity via feedback regulation, thereby promoting the conversion of saturated fatty acids (e.g., stearic acid) to MUFAs. OA, through activation of the AMPK/PGC‐1α pathway, upregulates TFAM expression, increasing mtDNA copy number and OxPhos capacity [36]. These data collectively underscore the pivotal role of SCD2 in maintaining lipid metabolism and OxPhos homeostasis in microglia. This was also confirmed by in vivo experiments: overexpression of AAV‐SCD2 in the hippocampus not only attenuated microglial pro‐inflammatory activation but also improved synaptic marker expression and neuronal survival, which ultimately alleviated cognitive dysfunction. It has been shown that SCD inhibitors (SCDi) lead to a reduction in synaptic proteins in AD models [23]. Hippocampal overexpression of TREM2 in mice fed a high‐fat diet resulted in a significant improvement in spatial memory (water maze) and recognition memory (novel object recognition) and in up‐regulated expression of synaptic proteins [37].

However, there are some limitations to this study. First, although SCD2 overexpression showed beneficial effects in our animal model, its translational potential and efficacy in humans require further verification [38]. Second, this study mainly focused on the effects of SCD2 on microglial metabolism and inflammation, but diabetic cognitive impairment is a complex, multifactorial disease, and future studies should consider more potential modulators and signalling pathways [39]. Moreover, a limitation of this study is the lack of genetic knockout models (e.g., microglia‐specific Scd2‐deficient mice), which would provide more direct evidence for the causal role of SCD2; future studies employing such models will help validate and extend our findings. Finally, the findings from BV2 microglia‐like cells should be interpreted with caution, as this immortalized line differs from primary microglia; further validation in primary cells is warranted. The present study revealed, for the first time, the critical regulatory role of SCD2 in diabetes‐associated cognitive dysfunction, which provides a new perspective for the development of therapeutic strategies targeting the metabolic homeostasis of microglial cells. Although there are some limitations, the present study provides an in‐depth understanding of the molecular mechanisms of diabetic cognitive impairment and offers an important reference for future clinical applications.

## 5 Conclusions

This study systematically elucidated the key role of SCD2 in DACD and its molecular mechanism. By integrating bioinformatics analysis, cellular and animal experiments, this study found that SCD2 expression was significantly down‐regulated in microglia under a diabetic state, which led to monounsaturated fatty acid metabolism disorders, impaired oxidative phosphorylation and abnormal accumulation of lipid droplets. Furthermore, SCD2 overexpression or OA supplementation significantly ameliorated diabetes‐associated cognitive impairment.

## Author Contributions

**Yang Yang:** conceptualisation, investigation, data curation, formal analysis, funding, writing – original draft, writing – review and editing. **Juan He:** conceptualisation, investigation, data curation, formal analysis, writing‐original draft, writing – review and editing. **Jing Zheng:** conceptualisation, formal analysis, methodology, writing – original draft, writing – review and editing. **Bing Shao:** software, methodology, writing – review and editing. **Lixin Shi:** conceptualisation, investigation, project administration, resources, supervision, writing – review and editing. **Miao Zhang:** conceptualisation, project administration, resources, supervision, writing – review and editing.

## Funding

This work was supported by Noncommunicable Chronic Diseases‐National Science and Technology Major Project (2025ZD0550700/2025ZD0550703), Key Advantageous Discipline Construction Project of Guizhou Provincial Health Commission in 2024, Guizhou Province Science and Technology Project for qian ke he zhongda [2025] 008 (KJT‐2025005‐02) to Miao Zhang, Doctor Start‐up Fund of Affiliated Hospital of Guizhou Medical University (gyfybsky‐2023‐02) to Yang Yang, The Foundation Project for Science and Technology of Guizhou Provincial Health Commission in 2026 (gzwkj2026‐598) to Yang Yang, and The Project of Researching and Educating Students and Research Feedback on Teaching at the Affiliated Hospital of Guizhou Medical University in 2025 (gyfykj‐2025‐y20) to Yang Yang.

## Ethics Statement

All experimental protocols have been reviewed and approved by the Animal Ethics Committee of The Affiliated Hospital of Guizhou Medical University (No. 2403228).

## Consent

The data used in this study have never been published before.

## Conflicts of Interest

The authors declare no conflicts of interest.

## Supporting information


**Figure S1:** Cell type annotation. (A) Expression of different cell markers in various cell clusters. (B) Heatmap of cell type scores for different cell clusters assessed by ScType.
**Figure S2:** Correlation analysis of SCD2 expression with oxidative phosphorylation. (A) Violin plot showing SCD2 expression across different cell types. (B) Correlation between OxPhos activity and all genes in microglia and oligodendrocytes, with the SCD2 gene highlighted. (C) Expression of CA3‐related marker genes across different cell types.
**Figure S3:** Expression of SCD2 in CA1, CA3 and DG regions of the hippocampus. IF staining was performed to detect SCD2 expression in the CA1, CA3 and DG subregions. Data are presented as the mean ± SD; *n* = 3. Statistical significance was determined by an unpaired two‐tailed Student's *t*‐test. *p < 0.05, **p < 0.01 versus Control.
**Figure S4:** Activation of microglia‐like cells by lipids in T2D mouse plasma. BV2 microglia‐like cells were treated with plasma from T2D mice. LRA was included in one treatment group. Experimental groups: CON, T2D plasma and T2D plasma + LRA. (A) ROS levels were measured by flow cytometry. (B) ATP levels were quantified using a commercial ATP assay kit. (C) ELISA was conducted to determine the concentrations of TNF‐α and IL‐6 in the culture supernatant. (D) WB was utilized to analyse the protein expression levels of YM‐1, Arg‐1 and CD206. Data are presented as the mean ± SD; *n* = 3. Statistical analysis was performed by one‐way ANOVA with Tukey's test (for normally distributed data) or the Kruskal–Wallis test with Dunn's test (for non‐normally distributed data). *p < 0.05, **p < 0.01, ***p < 0.001 versus CON/T2D plasma.
**Figure S5:** Impaired oxidative phosphorylation reduces the levels of mtDNA‐encoded transcripts for complexes I, III, IV and V. (A, B) qRT‐PCR and WB detection of TfamA level in microglia‐like cells after treatment with si‐TfamA. (C) qRT‐PCR detection of the levels of mtDNA‐encoded transcripts for complexes I, III, IV and V in microglia after treatment with T2D plasma + si‐TfamA. Data are presented as the mean ± SD; *n* = 3. Statistical analysis was performed by one‐way ANOVA followed by Tukey's post hoc test. *p < 0.05, **p < 0.01, ***p < 0.001 versus si‐NC/CON/T2D plasma + si‐NC.
**Figure S6:** Colocalisation of PLIN2 with Iba1 in the hippocampus. IF staining was performed to assess the colocalisation of PLIN2 with IBa1. Representative images show staining for PLIN2 (green) and Iba1 (red). Nuclei were counterstained with DAPI (blue). Scale bar = 50 μm. Data are presented as the mean ± SD; *n* = 3. Statistical analysis was performed by one‐way ANOVA followed by Tukey's post hoc test. *p < 0.05, **p < 0.01, ***p < 0.001 versus Control/T2D + AAV‐NC.
**Figure S7:** SCD2 overexpression reverses the effects of T2D on oxidative phosphorylation in primary hippocampal microglia. Primary hippocampal microglia were isolated from each experimental group. (A) OCR was measured using a kit. (B) Lactate accumulation was detected using a lactate assay kit. (C, D) ECAR was evaluated using a kit. (E) ATP content detection kit was employed to measure ATP levels. (F–J) ELISA was used to analyse the FFA, TAG, MGL, FC and CE levels. (K) WB was utilized to assess PLIN2 protein expression level. Data are presented as the mean ± SD; *n* = 3. Statistical analysis was performed by one‐way ANOVA followed by Tukey's post hoc test. *p < 0.05, **p < 0.01, ***p < 0.001 versus Control/T2D + AAV‐NC.
**Figure S8:** SCD2 overexpression improves diabetes‐related cognitive dysfunction. (A, B) Morris water maze test. Data are presented as the mean ± SD; *n* = 6. Statistical analysis was performed by one‐way ANOVA followed by Tukey's post hoc test. *p < 0.05, **p < 0.01, ***p < 0.001 versus Control/T2D + AAV‐NC.

## Data Availability

The data that support the findings of this study are available from the corresponding author upon reasonable request.
