# Supplementary material for: SCD2 Alleviates Diabetes‐Associated Cognitive Dysfunction by Improving Microglial Lipid Metabolism
Source: Cell Prolif. 2026 Apr 30;59(9):e70221. doi: 10.1111/cpr.70221 (PMC13325817; doi:10.1111/cpr.70221)
Supplement: Supplementary file 1 [file CPR-59-e70221-s001.docx]

**Supplementary Figure 1 Cell type annotation.** (A) Expression of different cell markers in various cell clusters. (B) Heatmap of cell type scores for different cell clusters assessed by ScType.

**Supplementary Figure 2 Correlation analysis of SCD2 expression with oxidative phosphorylation.** (A) Violin plot showing SCD2 expression across different cell types. (B) Correlation between OxPhos activity and all genes in microglia and oligodendrocytes, with the SCD2 gene highlighted. (C) Expression of CA3-related marker genes across different cell types.

**Supplementary Figure 3 Expression of SCD2 in CA1, CA3, and DG regions of the hippocampus**. IF staining was performed to detect SCD2 expression in the CA1, CA3, and DG subregions. Data are presented as the mean ± SD; n = 3. Statistical significance was determined by an unpaired two-tailed Student’s t-test. ^*^*p* < 0.05, ^**^*p* < 0.01 vs Control.

**Supplementary Figure 4 Activation of microglia-like cells by lipids in T2D mouse plasma.** BV2 microglia-like cells were treated with plasma from T2D mice. LRA was included in one treatment group. Experimental groups: CON, T2D plasma, and T2D plasma + LRA. (A) ROS levels were measured by flow cytometry. (B) ATP levels were quantified using a commercial ATP assay kit. (C) ELISA was conducted to determine the concentrations of TNF-α and IL-6 in the culture supernatant. (D) WB was utilized to analyze the protein expression levels of YM-1, Arg-1, and CD206. Data are presented as the mean ± SD; n = 3. Statistical analysis was performed by one-way ANOVA with Tukey’s test (for normally distributed data) or the Kruskal-Wallis test with Dunn’s test (for non-normally distributed data). ^*^*p* ˂ 0.05, ^**^*p* ˂ 0.01, ^***^*p* ˂ 0.001 **vs CON/ T2D plasma.**

**Supplementary Figure 5 Impaired oxidative phosphorylation reduces the levels of mtDNA-encoded transcripts for complexes I, III, IV, and V.** (A-B) qRT-PCR and WB detection of TfamA level in microglia-like cells after treatment with si-TfamA. (C) qRT-PCR detection of the levels of mtDNA-encoded transcripts for complexes I, III, IV, and V in microglia after treatment with T2D plasma+si-TfamA. Data are presented as the mean ± SD; n = 3. Statistical analysis was performed by one-way ANOVA followed by Tukey’s post hoc test. ^*^*p* ˂ 0.05, ^**^*p* ˂ 0.01, ^***^*p* ˂ 0.001 vs si-NC/ CON/ T2D plasma+si-NC.

**Supplementary Figure 6 Colocalization of PLIN2 with Iba1 in the hippocampus.** IF staining was performed to assess the colocalization of PLIN2 with IBa1. Representative images show staining for PLIN2 (green) and Iba1 (red). Nuclei were counterstained with DAPI (blue). Scale bar = 50 μm. Data are presented as the mean ± SD; n = 3. Statistical analysis was performed by one-way ANOVA followed by Tukey’s post hoc test. ^*^*p* ˂ 0.05, ^**^*p* ˂ 0.01, ^***^*p* ˂ 0.001 vs Control/ T2D+AAV-NC.

**Supplementary Figure 7 SCD2 overexpression reverses the effects of T2D on oxidative phosphorylation in primary hippocampal microglia**. Primary hippocampal microglia were isolated from each experimental group. (A) OCR was measured using a kit. (B) Lactate accumulation was detected using a lactate assay kit. (C-D) ECAR was evaluated using a kit. (E) ATP content detection kit was employed to measure ATP levels. (F-J) ELISA was used to analyze the FFA, TAG, MGL, FC, and CE levels. (K) WB was utilized to assess PLIN2 protein expression level. Data are presented as the mean ± SD; n = 3. Statistical analysis was performed by one-way ANOVA followed by Tukey's post hoc test. ^*^*p* < 0.05, ^**^*p* < 0.01, ^***^*p* < 0.001 vs Control/ T2D+AAV-NC.

**Supplementary Figure 8 SCD2 overexpression improves diabetes-related cognitive dysfunction.** (A-B) Morris water maze test. Data are presented as the mean ± SD; n = 6. Statistical analysis was performed by one-way ANOVA followed by Tukey’s post hoc test. ^*^*p* ˂ 0.05, ^**^*p* ˂ 0.01, ^***^*p* ˂ 0.001 vs Control/ T2D+AAV-NC.

**Revised Supplementary Figure 1**

**Revised Supplementary Figure 2**

**Revised Supplementary Figure 3**

**Revised Supplementary Figure 4**

**Revised Supplementary Figure 5**

**Revised Supplementary Figure 6**

**Revised Supplementary Figure 7**

**Revised Supplementary Figure 8**
