# Supplementary material for: SCD2 Alleviates Diabetes‐Associated Cognitive Dysfunction by Improving Microglial Lipid Metabolism
Source: Cell Prolif. 2026 Apr 30:e70221. Online ahead of print. doi: 10.1111/cpr.70221 (PMC13325817; doi:10.1111/cpr.70221)

**Supplementary Figure 1 Cell type annotation.** (A) Expression of different cell markers in various cell clusters. (B) Heatmap of cell type scores for different cell clusters assessed by ScType.

**Supplementary Figure 2 Correlation analysis of SCD2 expression with oxidative phosphorylation.** (A) Violin plot showing SCD2 expression across different cell types. (B) Correlation between OxPhos activity and all genes in microglia and oligodendrocytes, with the SCD2 gene highlighted. (C) Expression of CA3-related marker genes across different cell types.

**Supplementary Figure 3 Expression of SCD2 in CA1, CA3, and DG regions of the hippocampus**. IF staining was performed to detect SCD2 expression in the CA1, CA3, and DG subregions. Data are presented as the mean ± SD; n = 3. Statistical significance was determined by an unpaired two-tailed Student’s t-test. ^*^*p* < 0.05, ^**^*p* < 0.01 vs Control.

**Supplementary Figure 4 Activation of microglia-like cells by lipids in T2D mouse plasma.** BV2 microglia-like cells were treated with plasma from T2D mice. LRA was included in one treatment group. Experimental groups: CON, T2D plasma, and T2D plasma + LRA. (A) ROS levels were measured by flow cytometry. (B) ATP levels were quantified using a commercial ATP assay kit. (C) ELISA was conducted to determine the concentrations of TNF-α and IL-6 in the culture supernatant. (D) WB was utilized to analyze the protein expression levels of YM-1, Arg-1, and CD206. Data are presented as the mean ± SD; n = 3. Statistical analysis was performed by one-way ANOVA with Tukey’s test (for normally distributed data) or the Kruskal-Wallis test with Dunn’s test (for non-normally distributed data). ^*^*p* ˂ 0.05, ^**^*p* ˂ 0.01, ^***^*p* ˂ 0.001 **vs CON/ T2D plasma.**

**Supplementary Figure 5 Impaired oxidative phosphorylation reduces the levels of mtDNA-encoded transcripts for complexes I, III, IV, and V.** (A-B) qRT-PCR and WB detection of TfamA level in microglia-like cells after treatment with si-TfamA. (C) qRT-PCR detection of the levels of mtDNA-encoded transcripts for complexes I, III, IV, and V in microglia after treatment with T2D plasma+si-TfamA. Data are presented as the mean ± SD; n = 3. Statistical analysis was performed by one-way ANOVA followed by Tukey’s post hoc test. ^*^*p* ˂ 0.05, ^**^*p* ˂ 0.01, ^***^*p* ˂ 0.001 vs si-NC/ CON/ T2D plasma+si-NC.

**Supplementary Figure 6 Colocalization of PLIN2 with Iba1 in the hippocampus.** IF staining was performed to assess the colocalization of PLIN2 with IBa1. Representative images show staining for PLIN2 (green) and Iba1 (red). Nuclei were counterstained with DAPI (blue). Scale bar = 50 μm. Data are presented as the mean ± SD; n = 3. Statistical analysis was performed by one-way ANOVA followed by Tukey’s post hoc test. ^*^*p* ˂ 0.05, ^**^*p* ˂ 0.01, ^***^*p* ˂ 0.001 vs Control/ T2D+AAV-NC.

**Supplementary Figure 7 SCD2 overexpression reverses the effects of T2D on oxidative phosphorylation in primary hippocampal microglia**. Primary hippocampal microglia were isolated from each experimental group. (A) OCR was measured using a kit. (B) Lactate accumulation was detected using a lactate assay kit. (C-D) ECAR was evaluated using a kit. (E) ATP content detection kit was employed to measure ATP levels. (F-J) ELISA was used to analyze the FFA, TAG, MGL, FC, and CE levels. (K) WB was utilized to assess PLIN2 protein expression level. Data are presented as the mean ± SD; n = 3. Statistical analysis was performed by one-way ANOVA followed by Tukey's post hoc test. ^*^*p* < 0.05, ^**^*p* < 0.01, ^***^*p* < 0.001 vs Control/ T2D+AAV-NC.

**Supplementary Figure 8 SCD2 overexpression improves diabetes-related cognitive dysfunction.** (A-B) Morris water maze test. Data are presented as the mean ± SD; n = 6. Statistical analysis was performed by one-way ANOVA followed by Tukey’s post hoc test. ^*^*p* ˂ 0.05, ^**^*p* ˂ 0.01, ^***^*p* ˂ 0.001 vs Control/ T2D+AAV-NC.

**Revised Supplementary Figure 1**

**
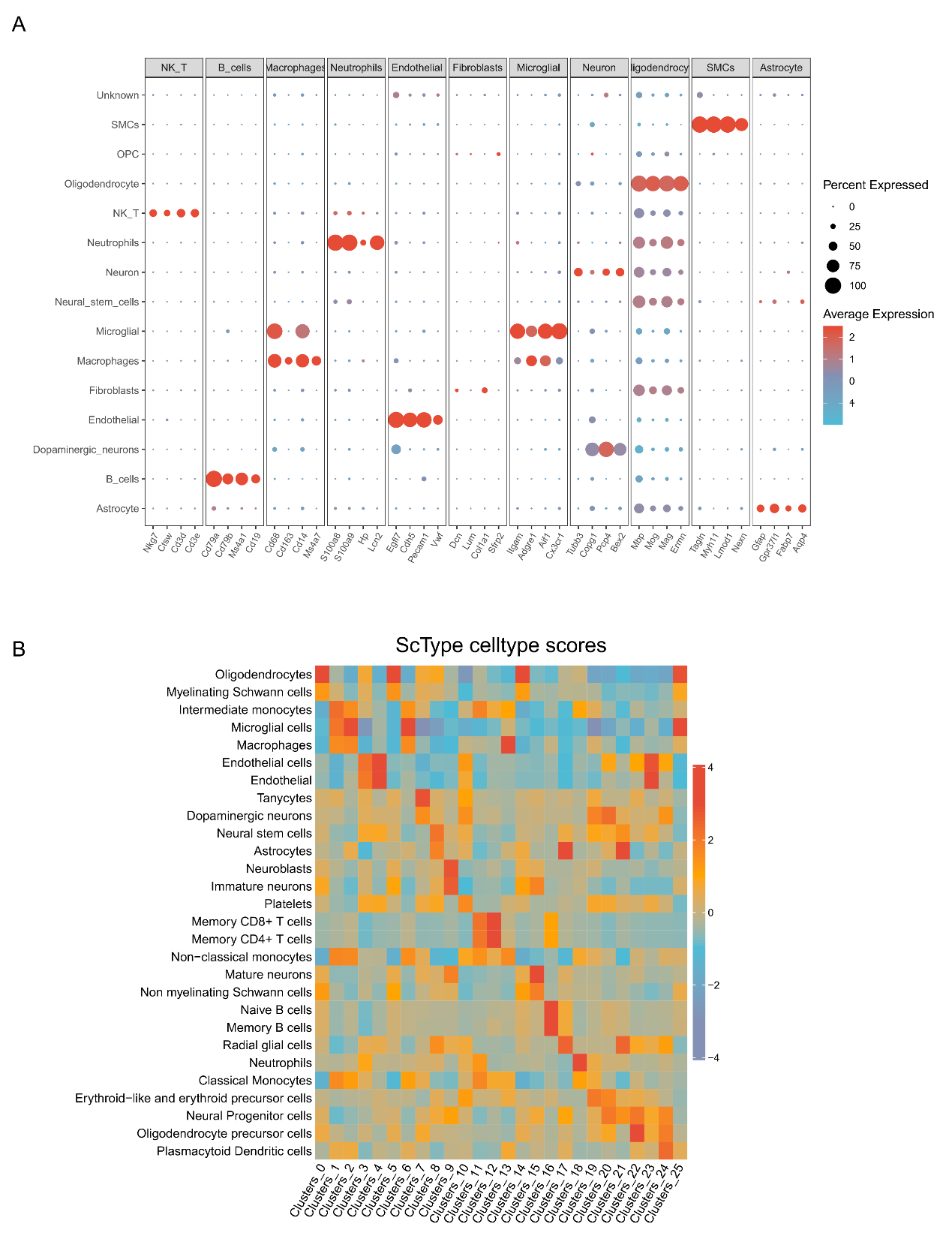
**

**Revised Supplementary Figure 2**


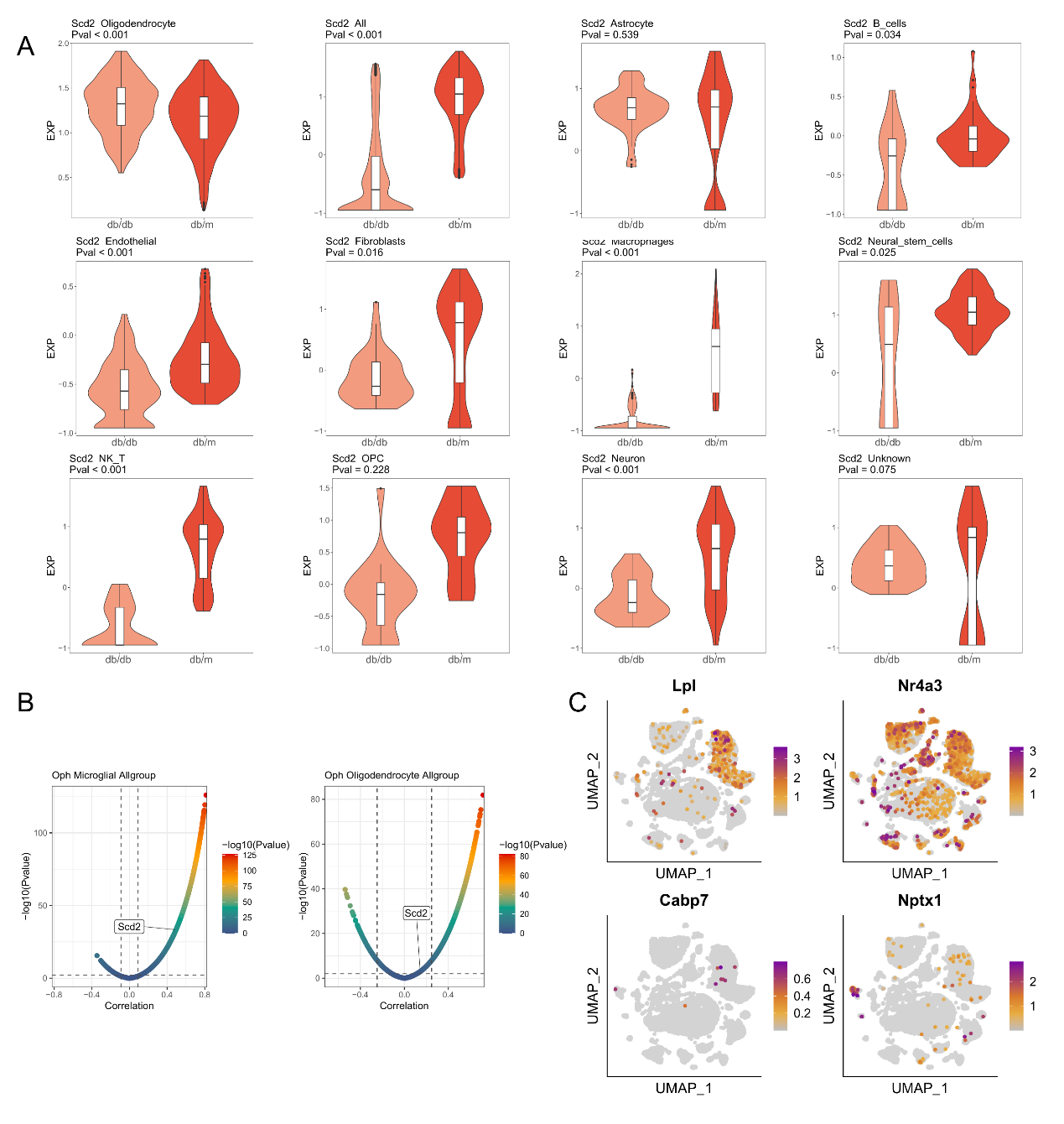


**Revised Supplementary Figure 3**


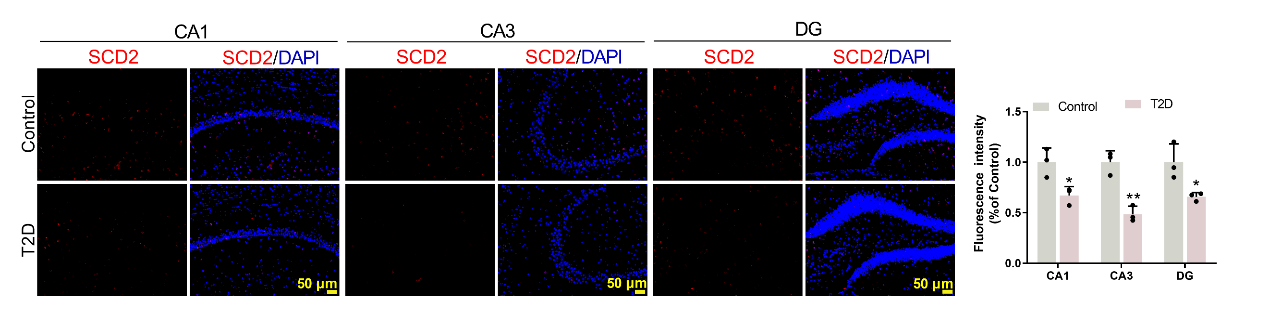


**Revised Supplementary Figure 4**

**
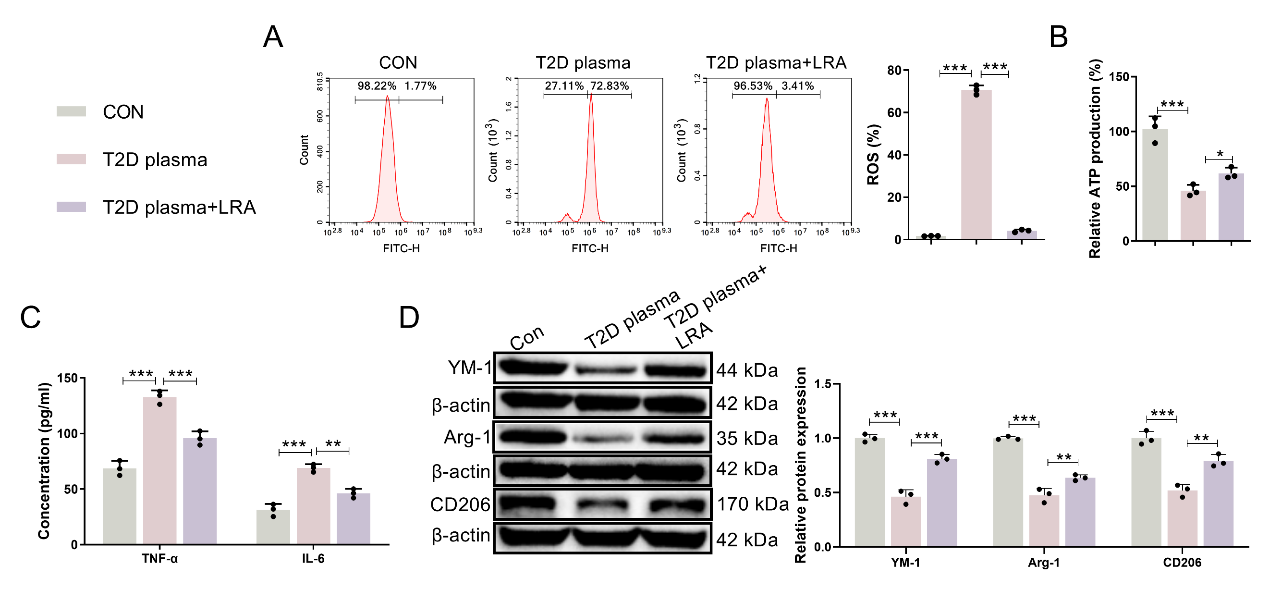
**

**Revised Supplementary Figure 5**


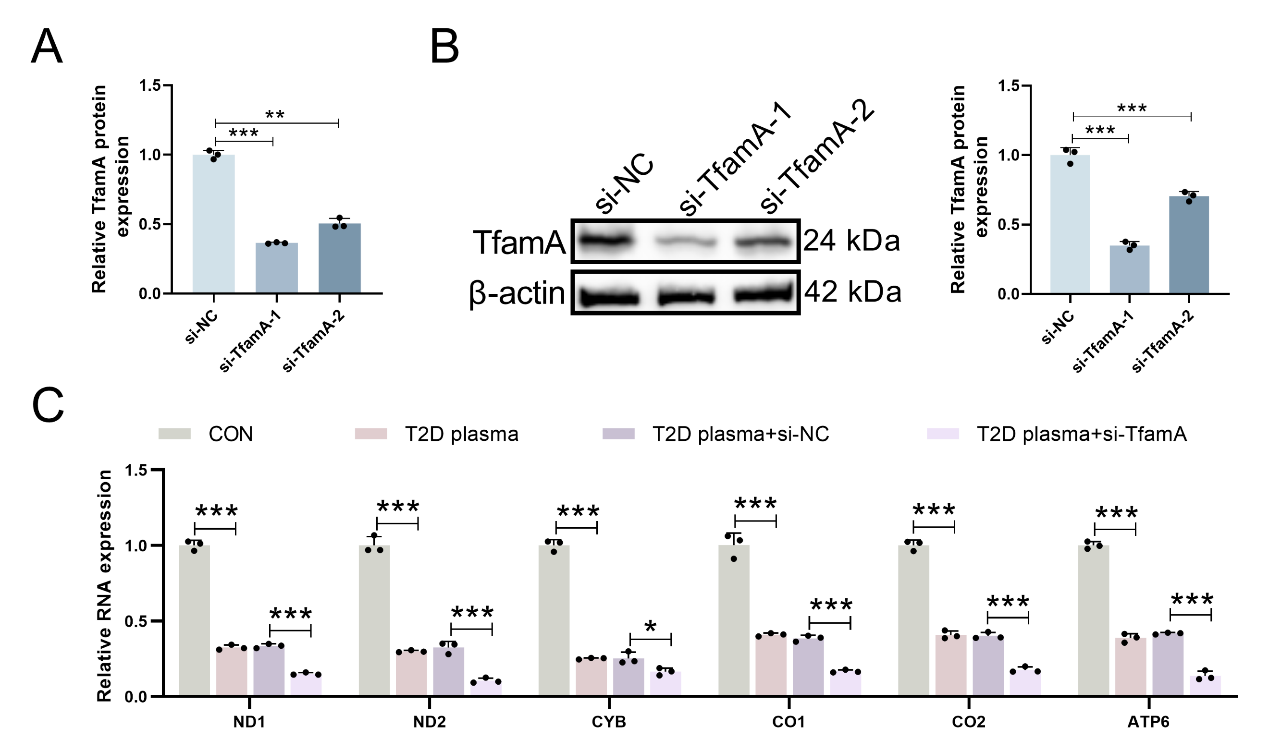


**Revised Supplementary Figure 6**


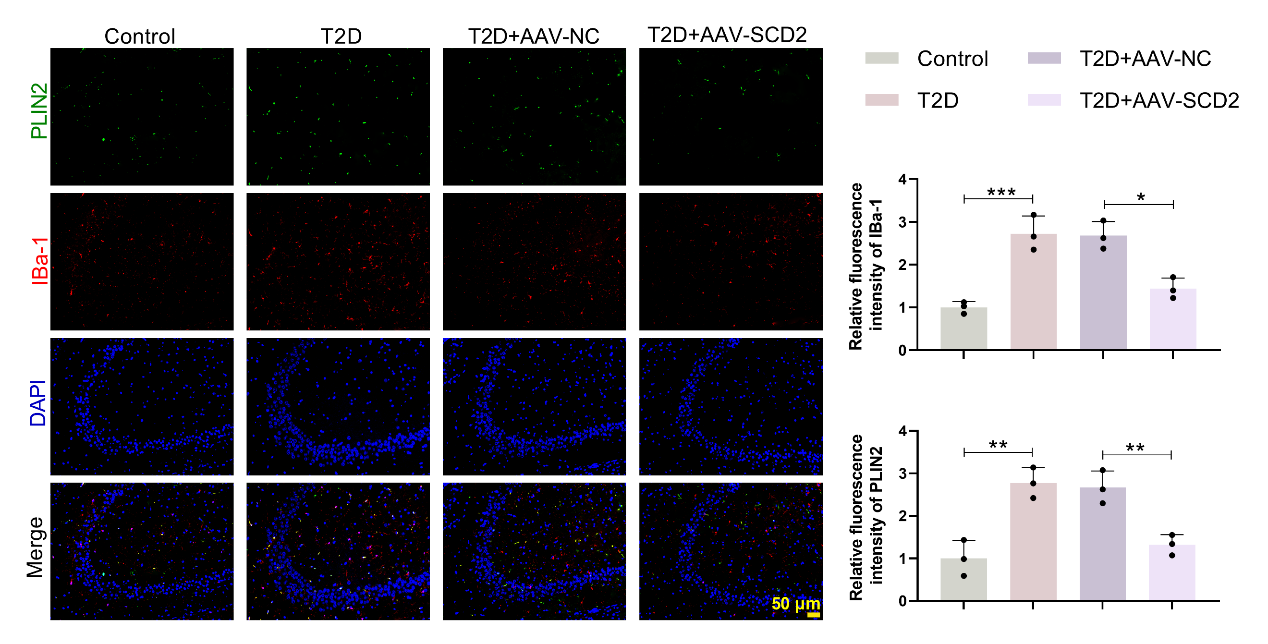


**Revised Supplementary Figure 7**


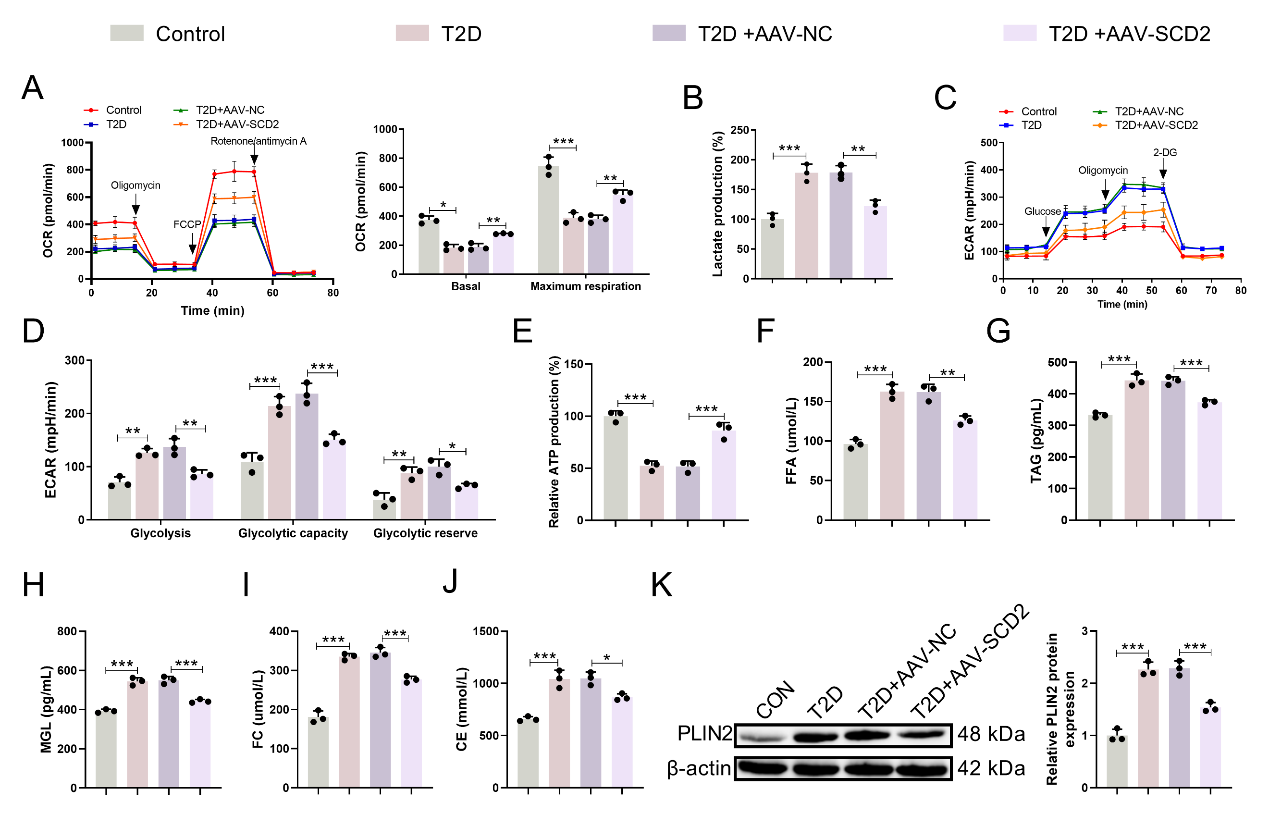


**Revised Supplementary Figure 8**


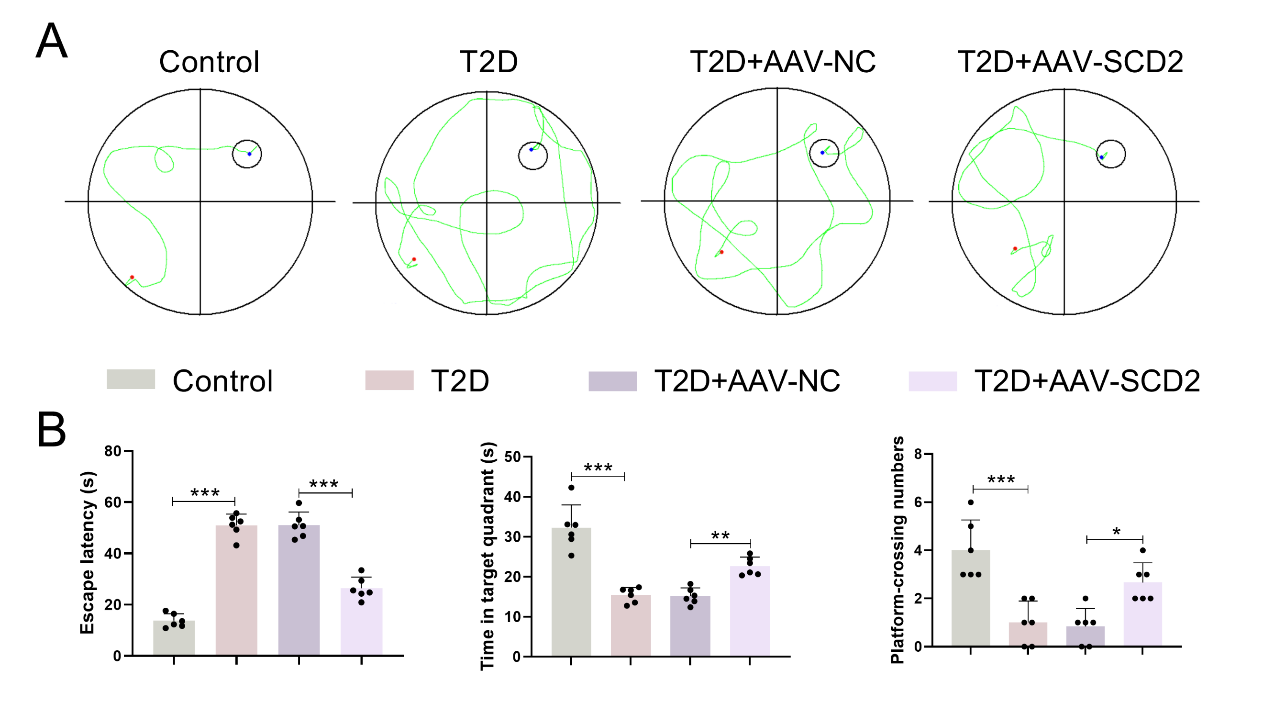

Supplement: Supplementary file 1 — Figure S1: Cell type annotation. (A) Expression of different cell markers in various cell clusters. (B) Heatmap of cell type scores for different cell clusters assessed by ScType. Figure S2: Correlation analysis of SCD2 expression with oxidative phosphorylation. (A) Violin plot showing SCD2 expression across different cell types. (B) Correlation between OxPhos activity and all genes in microglia and oligodendrocytes, with the SCD2 gene highlighted. (C) Expression of CA3‐related marker genes across different cell types. Figure S3: Expression of SCD2 in CA1, CA3 and DG regions of the hippocampus. IF staining was performed to detect SCD2 expression in the CA1, CA3 and DG subregions. Data are presented as the mean ± SD; n = 3. Statistical significance was determined by an unpaired two‐tailed Student's t‐test. *p < 0.05, **p < 0.01 versus Control. Figure S4: Activation of microglia‐like cells by lipids in T2D mouse plasma. BV2 microglia‐like cells were treated with plasma from T2D mice. LRA was included in one treatment group. Experimental groups: CON, T2D plasma and T2D plasma + LRA. (A) ROS levels were measured by flow cytometry. (B) ATP levels were quantified using a commercial ATP assay kit. (C) ELISA was conducted to determine the concentrations of TNF‐α and IL‐6 in the culture supernatant. (D) WB was utilized to analyse the protein expression levels of YM‐1, Arg‐1 and CD206. Data are presented as the mean ± SD; n = 3. Statistical analysis was performed by one‐way ANOVA with Tukey's test (for normally distributed data) or the Kruskal–Wallis test with Dunn's test (for non‐normally distributed data). *p < 0.05, **p < 0.01, ***p < 0.001 versus CON/T2D plasma. Figure S5: Impaired oxidative phosphorylation reduces the levels of mtDNA‐encoded transcripts for complexes I, III, IV and V. (A, B) qRT‐PCR and WB detection of TfamA level in microglia‐like cells after treatment with si‐TfamA. (C) qRT‐PCR detection of the levels of mtDNA‐encoded transcripts for complexes I, III, [file CPR-9999-e70221-s001.docx]
